# Real-time volumetric imaging of cells and molecules in deep tissues with Takoyaki ultrasound

**DOI:** 10.1038/s41467-026-72961-0

**Published:** 2026-05-21

**Authors:** Sunho Lee, Di Wu, Dina Malounda, Claire Rabut, Mikhail G. Shapiro

**Affiliations:** 1 Andrew and Peggy Cherng Department of Medical Engineering, California Institute of Technology, Pasadena, CA USA; 2 Division of Chemistry and Chemical Engineering, California Institute of Technology, Pasadena, CA USA; 3 Howard Hughes Medical Institute, Pasadena, CA USA

**Keywords:** Ultrasound, Molecular imaging, Synthetic biology, Nanoparticles, Microbiology

## Abstract

Acoustic contrast agents and reporter genes play a critical role in allowing ultrasound to visualize blood flow, map molecules and track cellular function in optically opaque living organisms. However, many advanced agents requiring high acoustic pressures have been imaged primarily in 2D, while biological phenomena of interest unfurl in three dimensions. Here, we introduce a method for efficient, dynamic imaging of contrast agents and reporter genes in 3D using multiplexed matrix array transducers. Our “Takoyaki” pulse sequence uses the simultaneous scanning of multiple focal points to excite contrast agents with sufficient acoustic pressure for nonlinear imaging while efficiently covering 3D space. We first characterize and benchmark Takoyaki imaging performance with gas vesicle contrast agents in vitro. Then we establish utility in cellular imaging by visualizing acoustic reporter gene expression in a mouse model of glioblastoma. Finally, we demonstrate real-time volumetric imaging by tracking the dynamics of fluid motion in mouse brain ventricles during and after intraventricular contrast injection. Takoyaki imaging enables a more comprehensive understanding of biological processes by providing spatiotemporal information in 3D within the constraints of accessible multiplexed matrix-array systems.

## Abstract (web summary)

Takoyaki imaging enables dynamic 3D ultrasound imaging of acoustic contrast agents and reporter genes using affordable multiplexed matrix arrays, visualising genetically-labeled brain tumours and tracking ventricular flow in mice in real time.

## Introduction

Ultrasound is a widely used technology for noninvasive biomedical imaging, valued for its deep penetration, high resolution, portability and safety. Recently, the traditional capabilities of ultrasound have been extended to large-scale visualization of biological systems and monitoring of cellular and molecular processes via volumetric 3D imaging^1^ and advanced contrast agents^2^. In addition to established synthetic microbubbles for vascular imaging^3^, emerging contrast agents such as nanobubbles^4^, phase-change nanodroplets^5^ and gas vesicles^6,7^ (GVs) make it possible to connect ultrasound with a larger variety of biological phenomena. GVs in particular have expanded the connection between ultrasound and biological function by serving as acoustic reporter genes^8–13^, molecular biosensors of enzymes and calcium^14,15^, and nanoscale contrast agents with enhanced circulation, extravasation, and biodegradation^16–21^.

In parallel, 2D transducer arrays have made it possible to acquire 3D volumes at a single transducer position, facilitating dynamic and longitudinal imaging^22–29^ without introducing the significant temporal asynchrony along one direction that accompanies using a mechanically sliding linear array probe. With decreasing costs due to advances in manufacturing and multiplexing, volumetric imaging is becoming increasingly practical and accessible.

Combining volumetric imaging with advanced contrast agents would greatly expand the capabilities of ultrasound in the biological and preclinical settings by enabling real-time visualization of cellular and molecular phenomena deep within opaque volumes in vivo, well beyond the <1 mm limit of conventional optical techniques for cellular and molecular imaging^30^. However, doing so carries significant challenges. Selective imaging of contrast agents relies on nonlinear pulse sequences that enrich contrast agent signals relative to background^31–34^. For example, GV imaging is typically performed with amplitude modulation (AM) methods^31,32^, which take advantage of reversible mechanical buckling of the GV under acoustic pressure to elicit nonlinear sound scattering distinct from mostly linear background^31,35,36^. Alternatively, BURST imaging detects GV expression with single-cell sensitivity by capturing distinctive signals produced transiently upon irreversible GV collapse^34^. Translating these approaches into 3D requires careful pulse sequence design within the constraints of array hardware.

Two classes of 3D imaging transducers compatible with financially accessible ultrasound systems (which typically have ≤ 256 electronic channels) are row-column arrays (RCAs) and multiplexed matrix arrays (MMAs). RCAs comprise two orthogonal, overlapping linear arrays with long elements^37–39^ and are capable of transmitting planar^27^ or sheet-like excitations^40^. Meanwhile, MMAs comprise a fully-sampled matrix probe (e.g., with 1024 elements) operated using a lower channel-count imaging system (e.g., with 256 channels) via an appropriate multiplexer (e.g., 4-to-1)^28,29,41^. While in principle MMAs provide greater flexibility than RCAs in their transmit and receive beam profiles, they carry their own constraints due to inter-element coupling and, in some commercial designs, the presence of gaps between subgroups of elements, requiring creative pulse sequence schemes.

Although some ultrasound sequences for MMAs have been developed^28,29,42^, most rely on unfocused beams that, along with transducer input voltage limits set to prevent probe damage, produce insufficient pressure ranges (<~1.3 MPa) for some nonlinear imaging schemes (e.g., >2.1 MPa for BURST^10^). Multi-line transmission (MLT)^43^ – which transmits multiple focused beams simultaneously – can span wider pressures without substantially compromising scanning speed, but prior MLT implementations for matrix arrays^44–46^ utilize combinations of variably angled transmissions with a full 1024-channel system, making them not directly compatible with MMAs.

**Fig. 1:**
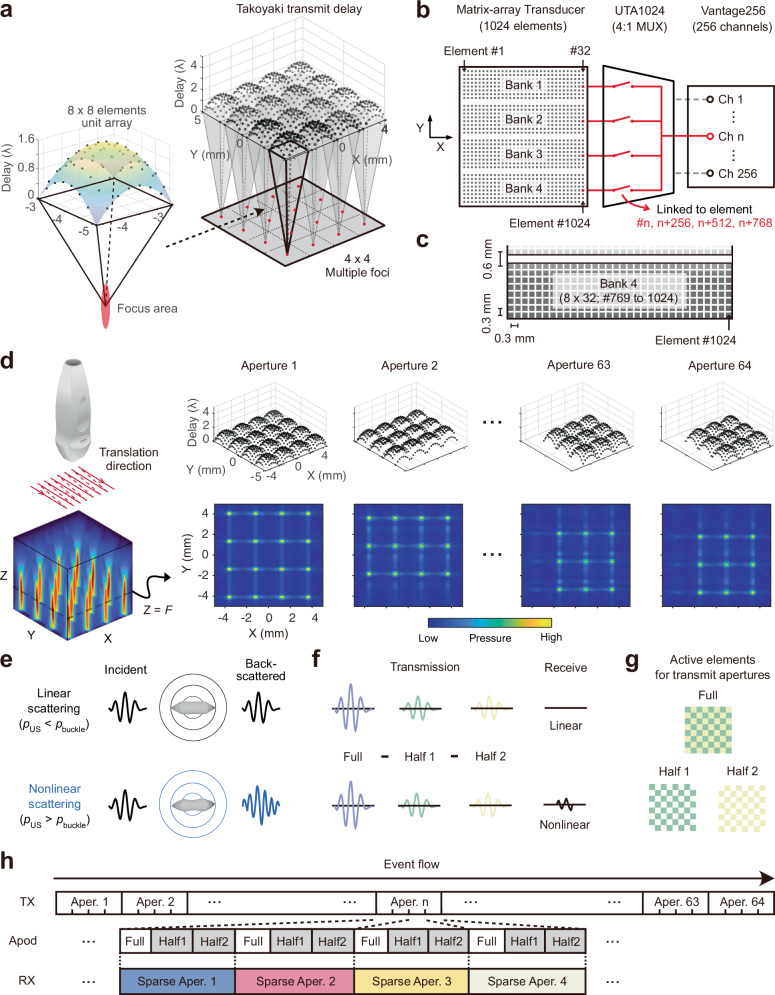
Takoyaki AM pulse sequence enables efficient scanning of a 3D volume. **a** Each unit array of 8 × 8 transducer elements generates focused ultrasound with a delay function concave in both X and Y, forming a cycle of Takoyaki functions. $\lambda$ denotes the wavelength. **b** Equivalent diagram of the multiplexing system. The 1024 matrix-array transducer elements are sectioned by three inactive rows (gaps) into four banks (8 × 32 elements each). A 4-to-1 multiplexer connects one system channel with four elements of the matrix-array probe. These elements (red) operate in parallel within each bank, spaced by eight active elements. A single system channel can signal any subset of the four parallel elements, but delays and apodizations must be equal. **c** Geometry of an element bank. **d** The Takoyaki delay and its focused beams move along the X and Y axes. The top and bottom rows show the delay functions and simulated pressure fields at Z = focus ($F$; 7 mm), respectively. **e** Nonlinear response of GVs to the pressure ($p$_US_) above the buckling threshold ($p$_buckle_). **f** In AM, the echoes of two half-amplitude transmissions are subtracted from that of full amplitude to distinguish between linear and nonlinear scatterers. **g** Two complementary checkerboard masks are used to generate half-amplitude transmissions. **h** Event flow of the Takoyaki AM sequence.

Here, we present a method for using MMAs to accomplish real-time volumetric imaging of acoustic contrast agents and reporter genes. Our “Takoyaki” paradigm uses all the elements of a square MMA to transmit axially directed focused ultrasound at multiple locations simultaneously using transmit delay functions resembling the Japanese street food Takoyaki (Fig. 1a). Implemented on a 1024-element (32 × 32) MMA with a center frequency of 15 MHz and a 256-channel ultrasound acquisition system (Fig. 1b, c), Takoyaki imaging operates within the constraints of parallel element operation, while efficiently scanning the entire underlying volume with acoustic pressure suitable for nonlinear contrast imaging (Fig. 1d). In this study, we implement and optimize Takoyaki imaging and reconstruction, characterize its performance in vitro, and demonstrate its superiority relative to alternative pulse sequences compatible with MMA. We then illustrate its applications in vivo by selectively imaging brain tumors expressing acoustic reporter genes and monitoring fluid transport in brain ventricles in mice in real time using injected GV contrast. Our study uses high-frequency (15 MHz) imaging to obtain high spatial resolution in imaging biomolecular and cellular events in the preclinical setting. Our results establish Takoyaki imaging as a high-performance method for volumetric imaging of more diverse nonlinear contrast agents that is compatible with affordable ultrasound instrumentation.

## Results

### The Takoyaki sequence allows efficient acquisition of 3D volumes

In the Takoyaki sequence, an MMA uses all 1024 elements to transmit axially directed focused ultrasound at multiple locations simultaneously, applying Takoyaki-shaped delay laws in which a hyperboloid function is periodically repeated across the 32 × 32 matrix array (Fig. 1a and Supplementary Fig. 1a). Each hyperboloid, corresponding to a single focus, involves an 8 × 8 array of transducer elements. The 8-element period in the Y axis satisfies the parallel element operation constraints imposed by the 4-to-1 multiplexing system connecting the MMA to the ultrasound acquisition platform (Fig. 1b, c). To scan the entire volume, the axial focused beams are translated by shifting the 8 x 8 element groups by one element along both the X and Y axes, reconstructing the regions corresponding to the foci to cumulatively cover the underlying 3D field of view (FOV), with 64 different transmit apertures (Fig. 1d).

In the AM paradigm, the nonlinear response of GVs to ultrasound (Fig. 1e) is isolated from linear background scattering by subtracting the echoes of two half-amplitude transmissions from those of a full amplitude transmission (Fig. 1f). For Takoyaki AM imaging, the half-amplitudes are generated by applying two complementary checkerboard masks to the transmit apertures (Fig. 1g). The ultrasound imaging sequence involves transmitting 12 pulses (resulting from 4 receive apertures across 3 AM modes) for each transmit aperture. Signals are then received by a complementary set of four sparse random apertures^41^, and these are coherently compounded across all 1024 elements to generate a single volume (Fig. 1h). With a pulse repetition frequency (PRF) of 4 kHz, the acquisition of Radio-Frequency (RF) data for each complete volume image is achieved in 192 ms, calculated from the 250 µs interval between transmissions multiplied by 64 transmit apertures with 12 transmissions each. This is at least 2.4 times faster than scanning with a single focused beam using the same Takoyaki AM parameters, which requires 469-1875 ms per volume (250 µs × 625 transmit apertures × 3 AM modes × 1-4 receive apertures), depending on the number of receive apertures used. To implement Takoyaki BURST imaging, the pressure is increased to collapse GVs, and GV signals are extracted by processing a temporal sequence of post-collapse frames.

Hardware considerations had to be addressed to put this sequence into practice. We implemented Takoyaki contrast imaging using a commercially available 1024-element (32 × 32) MMA with a center frequency of 15 MHz (wavelength ~ 100 µm) and an element pitch of 300 µm (lateral dimensions ~ 1 cm x 1 cm). Being three times larger than the wavelength, this pitch is expected to generate substantial grating lobes, degrading image quality. However, with Takoyaki contrast imaging and its short pulses, we expect the resulting off-focus pressure - including grating and side lobe contributions - to fall below the GVs’ nonlinear signal threshold (main-lobe-to-side-lobe pressure ratio > ~2.2 at Z = focal length; Supplementary Fig. 2), thereby suppressing grating and side lobe artifacts during molecular imaging. This large element width (~pitch size) also makes element directivity narrower; the sharper directivity of the elements helps alleviate receive crosstalk between the subapertures used in the Takoyaki sequence (Supplementary Fig. 3). A key benefit of the larger pitch is that it provides coverage of a 9-times larger FOV compared to wavelength-matched pitch for the same number of elements, while preserving high resolution crucial for cellular and biomolecular imaging. Additionally, as with most MMAs, element placement is not isotropic, and there are gaps between element banks in the Y dimension (Fig. 1b). We took these gaps into account in delay calculation for each transmit aperture.

### Takoyaki imaging provides high-quality isotropic contrast agent images

**Fig. 2:**
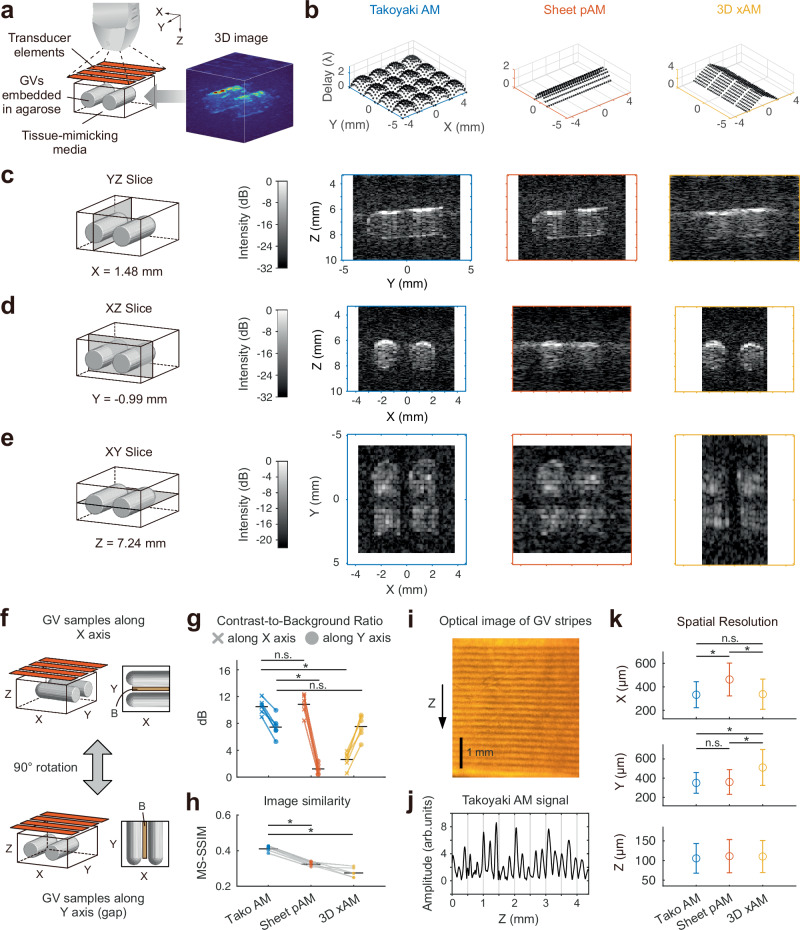
Takoyaki AM provides high-quality isotropic images of gas vesicles. **a** Experiment setup for imaging tissue-mimicking GV phantoms. The orange parallelograms represent the four banks of transducer elements. Each well in the phantom comprises a 5 mm-long cylinder and a hemisphere at one end, both with a 1 mm radius, with an interspace distance of 1 mm between neighboring wells. The axis of the wells was aligned with the Y axis, and the phantom location was adjusted so that its center was situated at Z = 7 mm. The 3D image of the phantom (OD = 6.5) acquired with Takoyaki AM is shown on the right. **b** Representative delay laws of Takoyaki AM (blue), Sheet-pAM (red), and 3D xAM (yellow). **c**–**e** Sliced phantom images acquired using different ultrasound sequences. The outer colored lines surrounding the sliced images indicate the maximum FOV. The color scale of the images is adjusted to match background levels across sequences. **f** Imaging GV phantoms aligned along the X and Y axes. The brown box (B) represents the interspace background. **g** CBRs of the volume images. Cross and circle marks correspond to phantoms aligned along the X and Y axes, respectively. *P*-values from top to bottom: 0.648, 2.75e-7, 4.64e-6, 0.946. **h** Similarity analysis. *P*-values from top to bottom: 1.04e-6, 7.90e-7. **i** Optical image of a phantom with GV stripes. **j** Takoyaki AM signal of GV stripes. **k** Spatial resolution based on the full-width half-maximum. *P*-values from top to bottom: 0.996, 1.47e-31, 6.47e-35, 5.63e-30, 3.84e-30, 0.942. Error bars represent ±std. Asterisks denote *p* < 0.01. Two-sided paired t-test for (**g**, **h)** (*N* = 6). Two-sided Mann-Whitney test for (**k**) (the number of detected peaks from 6 phantoms along each axis for Takoyaki AM, Sheet-pAM, and 3D xAM = X: 284, 302, 253; Y: 268, 348, 260; and Z: 1 × 10^4^, 1.5 × 10^4^, 1.3 × 10^4^, respectively). Takoyaki AM and Sheet-pAM transmissions were both focused at a depth of 7 mm. Source data are provided as a Source Data file.

To examine the characteristics of the Takoyaki sequence, we imaged tissue-mimicking phantoms containing GVs using Takoyaki AM and compared these images with those obtained using two other potential rapid imaging schemes compatible with MMA: sheet-pAM and 3D xAM (Fig. 2a, b). Sheet-pAM generates a sheet-like focus parallel to the XZ plane by cylindrically focusing ultrasound (Supplementary Fig. 1b, Methods). In contrast, 3D xAM, a dimensional extension of xAM^32^ (recently implemented on RCA^40^), emits two counter-propagating axisymmetric angled plane waves and images their bisector slices parallel to the YZ plane (Supplementary Fig. 1c, Methods). Both sequences form volume images slice-by-slice; on the contrary, the Takoyaki sequence forms volumes by reconstructing multiple lines in parallel. We did not consider Sheet-pAM and 3D xAM with alternate orientations (e.g., Sheet-pAM imaging YZ planes), nor did we explore combinations with these orthogonal implementations, since the gaps on the matrix array increase heterogeneity in Sheet-pAM’s pressure fields and disrupt the axisymmetry of the cross-propagating waves in 3D xAM. A sequence based on coherent plane-wave compounding was also not tested because its pressure levels (maximum ~1.3 MPa) are insufficient to robustly induce GV collapse for BURST imaging (>2.1 MPa^10^) and would be too low to drive AM-based imaging of certain GV types^10,15^ (Supplementary Fig. 4), especially when accounting for tissue attenuation in in vivo applications. Prior to the experiments, we empirically optimized the transmission amplitudes for each sequence to maximize AM signals without GV collapse, monitoring the images as we ramped the input voltage.

Takoyaki AM demonstrated the highest isotropic image quality of the three sequences (Fig. 2c–h). Sheet-pAM and 3D xAM images were relatively less sharp and more susceptible to horizontal smearing artifacts along one axis (X and Y, respectively), likely due to their slice-by-slice beamforming (Fig. 2c–e and Supplementary Movie 1). To evaluate the impact of probe direction on each sequence’s image quality, we imaged the same phantom aligned along the X axis or rotated to lie along Y (Fig. 2f). When the phantoms were aligned along the Y axis, all three sequences showed some signal dropout around Y = 0 (Fig. 2c, e and Supplementary Fig. 5), which we attribute to gaps in the matrix array and misalignment between transducer element banks^47^ (Supplementary Fig. 2). Comparing their contrast-to-background ratios (CBRs) with the background measured in the interspace between the two GV wells, the CBRs for Sheet-pAM and 3D xAM were each significantly compromised along one direction (Fig. 2g). In comparison, Takoyaki AM maintained high CBRs in both orientations. Takoyaki AM images also exhibited the highest similarity after phantom rotation, as indicated by the multi-scale structural similarity (MS-SSIM) index^48^, further supporting its superior isotropic imaging capabilities (Fig. 2h).

To assess the spatial resolution of Takoyaki imaging and the two alternative sequences, we imaged phantoms containing GVs in stripe patterns of sub-wavelength thickness generated by acoustic patterning^49^ (Fig. 2i, j and Supplementary Fig. 6). The measurements shared a similar tendency with the CBR analysis (Fig. 2k and Table 1): Takoyaki AM displayed the best spatial resolution for both lateral directions, while Sheet-pAM and 3D xAM showed poorer spatial resolutions in the X and Y axes, respectively, as expected because of their lack of transmit focusing in those directions. The axial resolution was similar across the different sequences. With ~330 μm lateral and ~105 μm axial resolution, Takoyaki imaging meets expectations based on element pitch and transmit wavelength.

**Table 1:** Comparison of Takoyaki AM, Sheet-pAM, and 3D xAM

Unit: *μ*m (mean ± std)	X resolution	Y resolution	Z resolution	# of transmissions
Takoyaki AM	333 ± 111	351 ± 107	105 ± 37.7	768
Sheet-pAM	463 ± 140	360 ± 128	111 ± 42.3	300
3D xAM	337 ± 129	511 ± 186	110 ± 40.8	204

All three imaging modes cover a partial FOV underneath the array (Fig. 2c–e). Takoyaki AM provides a FOV area (7.5 × 8.4 = 63 mm^2^) between those of 3D xAM (5.1 × 10.2 = 52.02 mm^2^) and Sheet-pAM (9.3 × 8.4 = 78.12 mm^2^). The number of transmissions required to cover these FOVs is 768 (64 transmit apertures × 3 AM × 4 receive apertures), 204 (17 transmit apertures × 3 AM × 4 receive apertures), and 300 (25 transmit apertures × 3 AM × 4 receive apertures) for Takoyaki AM, 3D xAM, and Sheet-pAM, respectively (Table 1).

As a result of its combined high isotropy, CBR, resolution and FOV, Takoyaki AM allows the matrix array probe to produce high-quality volumetric images of contrast agents. In addition to these performance advantages relative to alternative pulse sequences, Takoyaki AM’s two-dimensional parabolic focusing can, in principle, achieve higher pressures compared to 3D xAM and plane-wave-based sequences, making it more suitable for imaging modes that require exceeding specific pressure thresholds, such as BURST imaging.

### Takoyaki imaging enables volumetric visualization of genetically labeled brain tumors in mice

**Fig. 3:**
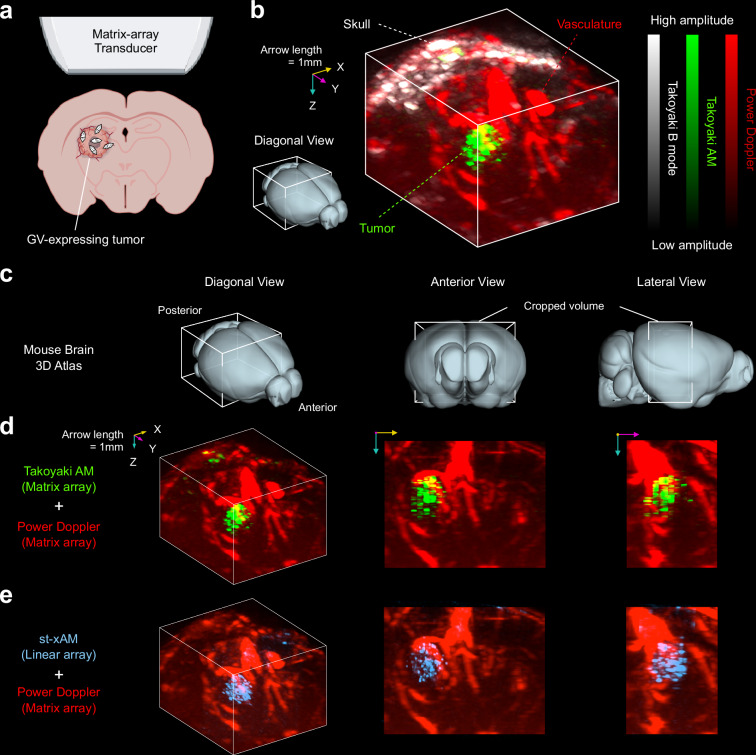
Takoyaki AM enables volumetric imaging of genetically labeled tumors in the mouse brain. **a** After tumor cell injection and growth, we induced GV expression and performed ultrasound imaging. Created with BioRender. Shin. G. (2026) https://BioRender.com/9sggxbg. **b** Overlay of Takoyaki B-mode (white), Takoyaki AM (green), and power Doppler images (red), acquired using an MMA transducer. **c** The Allen Mouse Brain Atlas^66^ in the top row illustrates brain orientation, with the white box indicating the imaging regions. **d** Takoyaki AM **e** Stacked xAM images obtained with a sweeping linear array probe (blue). For the anterior and lateral views, a half-cropped 3D image is used to exclude the skull side. Colored arrows represent the axes, with lengths of 1 mm.

To examine whether the Takoyaki ultrasound sequence can selectively image genetically labeled cells in 3D in vivo, we tested it using a brain tumor model. The ability to image brain tumors in situ is critical to understanding their growth dynamics, interactions with the neurovasculature and healthy brain tissue, and response to therapeutic interventions. Doing so in 3D with high temporal coherence would provide a more complete and accurate picture of these interactions and facilitate acquisitions that are less operator-dependent. We engineered U87 human tumor cells to express GVs as acoustic reporter genes and implanted them into mouse brains^50^ (Fig. 3a). These tumor cells were designed to form GVs and produce ultrasound contrast upon induction with the small molecule doxycycline. Following 6 days of tumor progression and a 2-day induction period, a portion of the skull (~4 mm anteroposterior x 8 mm lateral) was removed and Takoyaki AM images of the brain were acquired (Fig. 3b). During this imaging session, power Doppler images were also acquired with the MMA system, based on the sequence described in Chavignon et al. (2022)^29^, to provide vascular context to the tumor images, along with Takoyaki B-mode images for enhanced anatomical reference. For comparison, a 3D reconstruction of the tumors was generated by systematically translating a 15 MHz linear array probe (0.1 mm pitch) in 0.2 mm intervals on a stereotaxic instrument and artificially stacking the resulting 2D xAM^32^ images (denoted as st-xAM image), which is a gold standard linear array imaging method to visualize GVs.

Takoyaki AM successfully provided selective imaging of brain tumors, capturing their 3D volume and location inside the brain (*N* = 2; Fig. 3c, d and Supplementary Fig. 7). Aligning Takoyaki AM with power Doppler images enabled precise localization within the vascular anatomy (Fig. 3d and Supplementary Movie 2). While st-xAM achieved similar results with the expected higher lateral (X axis) resolution (Fig. 3e and Supplementary Movie 2), it required a much longer scanning time to acquire a similar imaging volume due to the need to physically translate the probe (>6 min). Even assuming automated robotic scanning with a 0.3-mm step size, this approach would take more than ~5 s (25 × 0.2-s movement intervals^51^), compared to just 192 ms for a frame of Takoyaki AM. As a result, the Takoyaki sequence is fundamentally less susceptible to motion artifacts and compatible with frame acquisition within motion-free windows such as inter-breath periods in mice (which occur at ~1 Hz), as may be needed in high-motion anatomical locations such as the abdomen. CBRs were 15.27 dB for Takoyaki AM and 15.22 dB for st-xAM, indicating comparable clarity. The spatial consistency between the two modes was high, though some minor regions (lateral view in Fig. 3d, e and Supplementary Fig. 7) detected in st-xAM were missed by Takoyaki AM, likely due to array signal gaps. The shallow region (3.4 to ~5 mm depth in Supplementary Fig. 7) was also not captured in Takoyaki AM, presumably because the focal zone begins around 5 mm (Supplementary Fig. 2; see also Supplementary Note 1). This region could be detectable with a shorter focal length. Overall, these findings confirm Takoyaki imaging’s capability to visualize brain tumors through GV expression in vivo.

### Takoyaki AM enables dynamic real-time imaging of nanoparticle transport in mouse brain ventricles

Takoyaki imaging’s ability to perform rapid volumetric scanning makes it suitable for observing dynamic processes within a volume while maintaining high intra-frame temporal coherence. Leveraging this feature, we explored the feasibility of real-time monitoring of GV dynamics within the mouse brain ventricles. The ventricles are filled with cerebrospinal fluid (CSF), whose circulation plays a vital role in clearing metabolic waste and transferring nutrients and drugs to brain tissue, especially during sleep^52–54^. Being able to visualize the flow of CSF and particles contained within it is critical for comprehending its normal biological function and involvement in various diseases^53,54^. In addition, understanding CSF transport can inform work on drug delivery to the brain, in which therapeutics such as viral vectors are administered into the ventricles to circumvent the blood-brain barrier^55,56^.

**Fig. 4:**
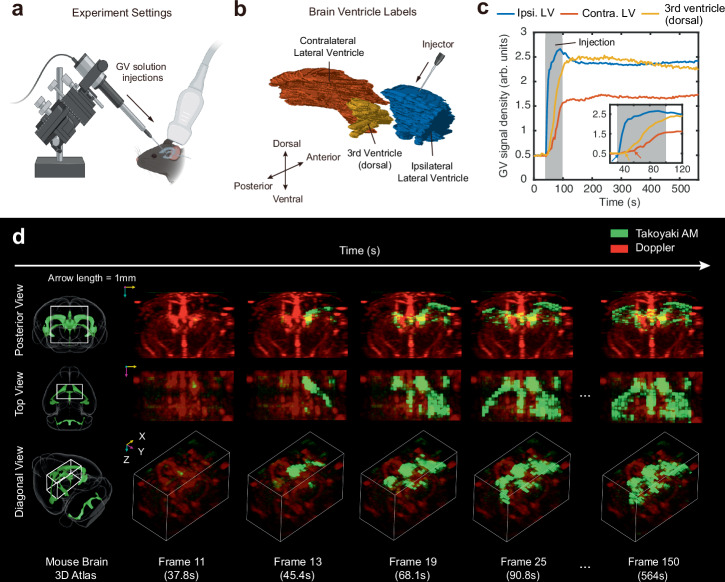
Takoyaki AM enables real-time dynamic imaging of nanoparticle transport within mouse brain ventricles. **a** Microinjection of GV solutions into the right LV. Created with BioRender. Shin. G. (2026) https://BioRender.com/9sggxbg. **b** Ventricle boundaries outlined based on GV signals. Blue, red, and yellow denote the ipsilateral LV to the injection site, contralateral LV, and third ventricle, respectively. Outlines were drawn manually based on AM images. Created with BioRender. Shin. G. (2026) https://BioRender.com/9sggxbg. **c** Temporal evolution of GV signal density. Injection commenced between Frame 11 and 12 (~40 s), and lasted for 60 s, as indicated by the gray shaded area. The arrows in the inset mark the start of the rapid signal increases for each ventricle. **d** Selected frames from stored real-time images. Takoyaki AM images (green) are overlaid on power Doppler images (red). The Allen Mouse Brain Atlas^66^ in the leftmost column illustrates brain orientations, with white bounding boxes indicating the image location. Colored arrows have a length of 1 mm along their corresponding directions. The sampling interval was approximately 3.8 s. Source data are provided as a Source Data file.

To demonstrate dynamic imaging of induced CSF flow, we injected GVs into one of the brain ventricles and followed their distribution in real time (Fig. 4a). While real-time imaging, we infused 4.5 $\mu$L of GV solution (1.6 nM) into the right lateral ventricle (LV) at a rate of 75 nL s^–1^ over 60 s. This rate yields an estimated flow speed below ~1.5 mm s^–1^ across most ventricular areas (Supplementary Note 2). Before, during and after the infusion, we continuously acquired volume images, choosing a frame rate of 0.26 Hz (1 frame per 3.8 s) and a PRF of 4 kHz (volume acquisition time = 192 ms) to capture the expected dynamics with sufficiently high temporal coherence for flows ≤ 1.57 mm s^–1^ (Supplementary Note 2) and allow real-time image processing and display. This frame rate can be accelerated to >5 Hz, given the 192 ms acquisition time, by deferring or accelerating image reconstruction and storage (currently ~2.8 s/frame and ~1 s/frame, respectively). Power Doppler images were acquired before the GV injection as an anatomical reference. To quantify the dynamics of GV transport across the ventricles, we outlined them (Fig. 4b and Supplementary Fig. 8a) and measured their AM signal density over time (Fig. 4c and Supplementary Fig. 8b).

During real-time monitoring, we successfully captured the ventricular system and the propagation of GVs across both LVs and the third ventricle (*N* = 2; Fig. 4d and Supplementary Fig. 8c; Supplementary Movie 3), clearly distinguishable from the vascular structures seen in Doppler images. Initial GV dispersion was observed in the LV ipsilateral to the injection site, followed by distribution to the third ventricle and then to the contralateral LV. The signal density graphs reflected the nanoparticle spreading dynamics observed during the real-time recording (Fig. 4c and Supplementary Fig. 8b). Rapid signal increases, corresponding to GV influx and spreading, appeared sequentially in the ipsilateral LV, third ventricle, and contralateral LV. These results demonstrate the ability of Takoyaki AM imaging to track a dynamic biological process in 3D, representing the first time to our knowledge that cerebrospinal fluid transport has been visualized with ultrasound. This experiment also took advantage of the thermodynamic stability of GVs, and their ability to persist under ultrasound exposure, allowing the imaging of a single non-replenished bolus of contrast agent over close to 20 min. Other studies using 2D imaging have shown the ability of GVs and GV-expressing cells to follow processes lasting hours to weeks^10,57,58^.

### Takoyaki BURST enables higher-sensitivity volumetric imaging in gap areas and deeper regions

**Fig. 5:**
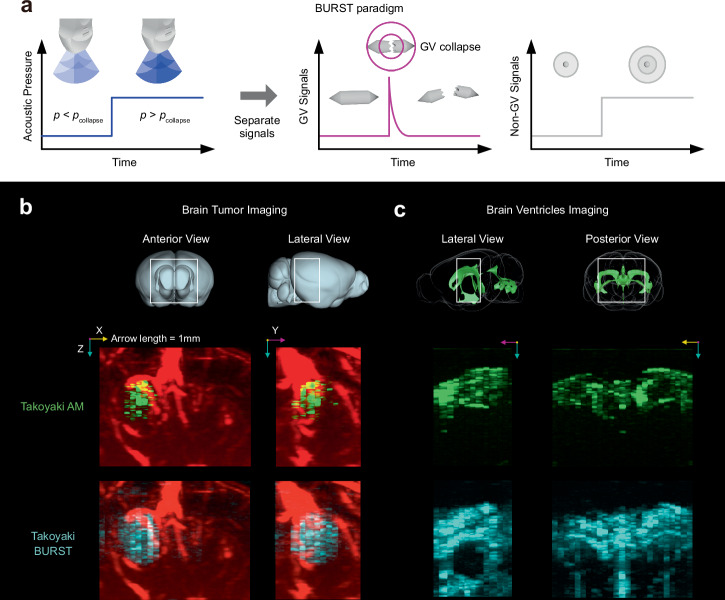
Takoyaki BURST enables higher-sensitivity volumetric imaging in gap areas and deeper regions. **a** BURST paradigm. Application of pressure exceeding the collapse threshold ($p$_collapse_) induces GV collapse, leading to strong transient GV signals, while non-GV signals remain constant. The two signals are separated after acquisition through processing. Comparison between Takoyaki AM (green) and BURST (cyan). The Allen Brain Atlas^66^ in the top row illustrates brain orientations, with white bounding boxes indicating the imaging regions. Colored arrows representing axes are scaled to a length of 1 mm along their respective directions. **b** Anterior and lateral views of a brain tumor. Power Doppler images (red) are overlaid. **c** Lateral and posterior views of brain ventricles.

In addition to its capacity for AM imaging, the Takoyaki sequence is well suited for ultrasensitive imaging using BURST^34^, which requires higher transmit pressure to irreversibly collapse GVs and isolate the resulting strong, transient signal. To validate its efficacy, we used the Takoyaki BURST sequence (Fig. 5a) to acquire images of brain tumors and ventricles following the completion of the corresponding AM imaging sessions.

In brain tumor imaging, our Takoyaki BURST sequence demonstrated enhanced visualization capabilities, effectively capturing signals from gap areas in the array and deeper regions that were difficult to discern with AM alone (Fig. 5b and Supplementary Fig. 9a; Supplementary Movie 4). This improved depth sensitivity was especially beneficial for imaging brain ventricles, where BURST clearly visualized the ventral portion of the third ventricle and LVs (Fig. 5c and Supplementary Fig. 9b; Supplementary Movie 4), whereas AM imaging was limited to dorsal regions. While optimization of GV expression levels, injected concentration, and focal beam parameters could enhance Takoyaki AM performance, these results show that the 3D BURST approach offers a robust way to maximize signal detection across the volume when a single snapshot is sufficient.

## Discussion

In this work, we introduced the Takoyaki volumetric ultrasound sequence, an imaging approach that enables efficient capture of contrast agent and reporter gene signals in 3D using the emerging class of commercially available MMA transducers. Using a repeated hyperbolic delay pattern that adheres to the parallel element constraints of an MMA, the Takoyaki sequence maximally utilizes the available transducer elements to generate focused transmissions at multiple locations simultaneously. This provides efficient scanning of the underlying volume while applying sufficient transmit pressure to elicit robust nonlinear contrast signals. Using this approach to implement nonlinear AM and BURST imaging, we showed that Takoyaki imaging can effectively capture the 3D distribution of specific cells (brain tumors) and biomolecules (gas vesicles) inside opaque tissues such as the mouse brain. Furthermore, Takoyaki AM enables dynamic 3D imaging, revealing, to our knowledge, for the first time, the real-time transport of nanoparticles across mouse brain ventricles. Meanwhile, Takoyaki BURST provided highly sensitive and comprehensive 3D snapshots of contrast agent distribution. Compared to alternative pulse sequences we implemented on MMA, Takoyaki imaging provided the best combination of sensitivity and isotropy.

Given the relative accessibility of 256-channel ultrasound scanners and MMAs, and the global availability of GV-based genetic constructs^10,59^, we anticipate the use of Takoyaki imaging in a wide range of biological research and biomedical applications. With currently ongoing rapid developments in GVs, these applications will not be limited to what we demonstrated in this study, but could in the future include intracellular biosensing of enzymes or calcium^14,15^, gastrointestinal inflammation sensing^11^, multiplexed biomolecular ultrasound^12^, and monitoring of T-cell therapy^13^. In addition, Takoyaki AM should be applicable to any other ultrasound contrast agent with nonlinear pressure responses, such as microbubbles^2^ and nanobubbles^4^, and extensible to other imaging modes such as pulse inversion. Where higher pressures are needed using the same amount of power, they could be amplified by distributing power across fewer banks of the array during transmission. For instance, a (4 × 1)—form Takoyaki BURST, using only one out of four parallel elements, could generate approximately double the pressure of the original (4 × 4)-form on the Verasonics Vantage 256.

The presented Takoyaki approach can be improved with future modifications. The heterogeneity of pressure fields caused by transducer element misalignment and gaps leads to loss of signal in certain regions of the FOV. This pressure heterogeneity could be mitigated by adjusting the apodization amplitude for each transmission and/or calibrating the element positions^47^. Furthermore, imaging depth could be improved by implementing multi-depth delays and managing contrast agent concentrations to avoid shadowing. Likewise, as needed, aberrations in the medium could be corrected computationally^60^. Dynamic imaging with Takoyaki AM (192 ms per frame) can already be fast enough for many biological phenomena (<~2.5 Hz), but its frame rate is currently slowed down by data processing and storage steps when real-time display is required. Online reconstruction could be accelerated by implementing it on graphical processor units. In theory, Takoyaki AM could ultimately reach an acquisition speed of 17.4 ms per frame for a 1-cm imaging depth, considering the minimum pulse-echo time of flight. The timescale of target phenomena appropriate for Takoyaki AM imaging, therefore, would be ~25 Hz (Nyquist sampling), ideally accompanied by optimized code to enable real-time visualization. With these enhancements and options, Takoyaki imaging will come in multiple flavors to satiate any hunger for 3D contrast-enhanced, biomolecular and cellular ultrasound.

## Methods

### Ethical approval

All animal experiments were conducted under protocols approved by the Institutional Animal Care and Use Committee (IACUC) at the California Institute of Technology. Mice were housed in a controlled facility with a 13-h light/11-h dark cycle (lights on from 6 am to 7 pm), 71-75 °F ambient temperature, and 30–70 % humidity.

### Ultrasound acquisition system

The 15 MHz matrix array probe (Vermon) comprises 32 x 32 transducer elements with a 0.3 mm pitch size. The elements are organized into four active banks, each containing 8 × 32 elements, separated by three inactive rows (Fig. 1b, c). The matrix probe was connected to a programmable ultrasound system (Verasonics Vantage 256) via a multiplex adapter (Verasonics UTA 1024-MUX). Each system channel can control up to four elements ($i$, $i$+256, $i$+512, $i$+768) in parallel, where $i\in${1, 2, …, 256}, mandating that elements at equivalent positions in each bank share the same delay and apodization settings. A waveform with a 15.625 MHz center frequency, 0.67 duty cycle, and 2 half-cycle periods was used, unless specified otherwise. All ultrasound sequence scripts were written and implemented via MATLAB 2022a (MathWorks). All ultrasound images were reconstructed with the Verasonics Vantage (version ≥ 4.4.0) built-in beamformer, with the sensitivity cut-off = 0.6. The sample mode was ‘NS200BW’, which resulted in the sampling frequency of 62.5 MHz.

### Takoyaki AM/B-mode sequences

The Takoyaki-like delays use unit arrays of (8 x 8) transducer elements to create focused ultrasound beams. The first transmission aperture, representing the basic form in the Takoyaki sequence, consists of 16 unit arrays arranged in a 4 × 4 grid, forming a full aperture of (32 × 32) elements (Fig. 1a). Throughout the transmission session, this basic delay pattern is translated across the entire transducer elements. There are 8 possible translations along both the X and Y axes—in total, 64 different apertures. This translation is akin to moving the matrix array probe across the XY plane while maintaining the basic Takoyaki delay pattern (Fig. 1d). Note that changes in gap locations within the unit arrays were considered in the delay calculations. The imaging regions for each transmission are basically cuboids (Frustum-shaped volumes of 0.3 mm × 0.3 mm × depth range) centered at each focus; however, for the 2nd and 8th translations along the Y direction, the Y width of the cuboids was set to 0.45 mm instead of 0.3 mm to compensate for the longer jump caused by gaps between transducer elements, resulting in the cuboids not being exactly centered at the foci. We inactivated elements on the unit arrays that are truncated after translating the delay function. Although this leads to a change in the number of parallel elements used and thus variations in pressure levels for different aperture types (8 apertures use all 4 parallel elements, and the others use 3 parallel elements), we found that for our matrix array probe, this inactivation gave less variance in pressure levels at the focal area across different aperture types compared to not performing such inactivation.

In the AM sequence, a full-amplitude ultrasound transmission is followed by two half-amplitude transmissions, and the received signals corresponding to the half-amplitudes are subtracted from that of the full-amplitude (Fig. 1f). Conversely, B-mode imaging uses only the full-amplitude transmission. For the Takoyaki AM sequence, the half-amplitude ultrasounds are generated by applying two complementary checkerboard masks to the transmission apodization (Fig. 1g). For the receive apertures, a complementary set of four sparse random apertures^41^ was used. Each transmission aperture undergoes an AM sequence (three transmissions) for each of the four receive apertures, and the received signals are coherently compounded (Fig. 1h). Data transfer and reconstruction occur once RF data for all 64 transmit apertures are collected. During the reconstruction, the cuboid imaging regions corresponding to each transmission aperture are beamformed in parallel based on the combined received signals from the four receive apertures. After the reconstruction, the image is visualized and then stored. The focal length was 7 mm. The reconstruction depth of interest was set from 3.3 mm to 10 mm, with a voxel size of half wavelength ($\lambda$/2 = 49.3 µm) for all sides. The pulse repetition frequency (PRF) was 4 kHz. Pressure field simulations were performed using the TXPD tool within the Verasonics Vantage software.

### Takoyaki BURST sequence

In the BURST paradigm^34^, a pressure higher than the collapse threshold of GVs is applied following a lower pressure, and distinctive transient GV signals are then extracted from the background (Fig. 5a). We used two different sequences to obtain the BURST images.

First, we developed an independent script that implements the BURST scheme without combining signals across complementary receive apertures. This script is fundamentally based on the event flow of Takoyaki B-mode but employs only one receive aperture per transmit aperture type. Additionally, the receive aperture for each transmit aperture varies randomly and is not identical. The number of frames captured at low pressure (Voltage = 1.6 V, Peak positive pressure = 60 kPa) was set to 2, while at high pressure (Voltage ≥ 27 V, Peak positive pressure ≥ 2 MPa), it was set to 28. Unlike the live imaging (Takoyaki AM) script, which reconstructs images after obtaining the RF data for each frame, this BURST script saves the RF signals for all frames first and then reconstructs the images later. IQ data are accumulated across frames. The PRF and time interval between frames were 4 kHz and 20 ms, respectively. The focal length was 7 mm. The number of half cycles was set to 3. This script was used for brain tumor experiments.

Alternatively, for brain ventricle imaging experiments, we utilized the live imaging script of the Takoyaki sequence with a high-pressure setting (Voltage ≥ 28 V). After increasing the pressure, ten frames were stored and then processed to extract the GV signals. The same parameters used in the Takoyaki AM imaging were applied.

### Sheet-pAM and 3D xAM

In Sheet-pAM, each transmission involves an array of (32 × 8) transducer elements, generating a sheet-like focus parallel to the XZ plane (Supplementary Fig. 1b). During imaging, a (9.6 mm × 0.3 mm × depth range) cuboid region centered at the focus is reconstructed based on the combined received signals from the four receive apertures used in Takoyaki AM. The focus moves across the entire array along the Y axis through 25 sequential translations. To compensate for larger jumps due to gaps in the matrix array, the same method used in Takoyaki AM was applied. The focal length was 7 mm.

For 3D xAM, each transmission utilizes an array of (16 × 32) elements. Each half (8 × 32) emits an angled plane wave that is parallel to the Y axis and symmetric with respect to the other (Supplementary Fig. 1c). The bisector slice (0.3 mm × 10.5 mm × depth range) parallel to the YZ plane is reconstructed, similar to xAM with a linear array^32^, based on the combined received signals from the four receive apertures used in Takoyaki AM. The X-waves are sequentially translated 17 times along the X axis. An angle of 6° relative to the matrix array probe surface was chosen for the X-waves, as it showed the best CBR across a range of tested angles under identical voltage conditions.

Both AM sequences share the same event flow structure as Takoyaki AM, but 3D xAM differs in how the half-amplitude ultrasound is generated. In 3D xAM, the half-amplitude ultrasound is produced by using only each half (8 × 32) elements, whereas Sheet-pAM employs checkerboard masks on its apertures. The depth range and other parameters were identical to those used in Takoyaki AM. Pressure field simulations were performed using the TXPD tool within the Verasonics Vantage software.

### BURST processing algorithm

BURST signals are traditionally processed using the temporal template unmixing algorithm^34^, which assumes that the peaks of GV signals only appear in the frame immediately following an increase in pressure. For example, the template vector for GV signals is defined as ${{{{\bf{u}}}}}_{g}={\left[010000\right]}^{T}$, when the first frame corresponds to low pressure and the subsequent frames to high pressure. In contrast, non-GV signals remain constant after increasing pressure, which is represented by the template vector ${{{{\bf{u}}}}}_{s}={\left[011111\right]}^{T}$. However, in our cases, we observed that GV collapse occurred over multiple frames, seemingly progressing towards deeper areas, possibly due to a shielding effect. Moreover, since we accumulated IQ data across frames, the background signal did not remain at a constant level but showed an increasing trend. Therefore, we used alternative methods for processing BURST signals instead of relying on the template unmixing algorithm.

Inspired by Demené et al.^61^, we took advantage of Singular Value Decomposition (SVD) to filter out coherent background signals from GV signals. Let ${{{\rm{S}}}}\in {{\mathbb{R}}}^{M\times N}$ denote the spatiotemporal matrix formed by reshaping the ${N}$ frames (4D matrix) into a 2D matrix. Then, SVD decomposes ${{{\rm{S}}}}$ as follows

1 $${{{\rm{S}}}}={{{\rm{U}}}}\Sigma {{{{\rm{V}}}}}^{*}={\sum}_{i=1}^{r}{{{{\bf{u}}}}}_{i}{\sigma }_{i}{{{{\bf{v}}}}}_{i}^{*}$$

where ${{{{\bf{u}}}}}_{i}$ is the $i$^th^ column of the matrix ${{{\rm{U}}}}$, ${{{{\bf{v}}}}}_{i}$ is the $i$^th^ column of the matrix ${{{\rm{V}}}}$, ${\sigma }_{i}$ is the $i$^th^ singular value, and $r$ is the rank of ${{{\rm{S}}}}$. SVD can be effective in our context, where the exact trend of the background template vector is uncertain, and there is minimal movement across frames. This is because the Eckart-Young theorem^62^ guarantees that the multiplication of the first components of SVD

2 $${{\rm{S}}}_{1}={{{{\bf{u}}}}}_{1}{\sigma }_{1}{{{{\bf{v}}}}}_{1}^{*}$$

provides the best rank-1 approximation of the spatiotemporal matrix ${{{\rm{S}}}}$. Given that the background signals can be interpreted as a spatial vector multiplied by a global temporal vector, subtracting ${{{{\rm{S}}}}}_{1}$ from ${{{\rm{S}}}}$, denoted as ${{{\rm{S}}}}-{{{{\rm{S}}}}}_{1}$, would effectively remove the background (linear scatterers), assuming that GV signals are sufficiently localized. Filtering out the first components of SVD can be expressed as

3 $${{{{\rm{S}}}}}_{F}={{{\rm{S}}}}-{{{{\rm{S}}}}}_{1}={\sum}_{i=2}^{r}{{{{\bf{u}}}}}_{i}{\sigma }_{i}{{{{\bf{v}}}}}_{i}^{*}={{{\rm{U}}}}\Sigma {{{{\rm{I}}}}}_{2:{{{\rm{r}}}}}{{{{\rm{V}}}}}^{*}$$

where ${{{{\rm{I}}}}}_{2:{{{\rm{r}}}}}\in {{\mathbb{R}}}^{r\times r}$ is a modified identity matrix with its first diagonal element set to zero. Although additional noise reduction may be achievable by discarding some of the last SVD components, we chose not to implement it for the sake of simplicity. Assuming successful background removal from the spatiotemporal images and no other sources of strong fluctuations,

4 $${{\mathrm{BURST}}}\;{{\mathrm{image}}}=	{\max}_{{{\mathrm{along}}}\;{{\mathrm{temporal}}}\;{{\mathrm{axis}}}} {{\mathrm{S}}}_{F} \ \\=	 {\max}_{{{\mathrm{along}}}\;{{\mathrm{temporal}}}\;{{\mathrm{axis}}}}{{\mathrm{U}}}\Sigma {{\mathrm{I}}}_{2:{{\mathrm{r}}}}{{\mathrm{V}}}^{*}$$

could properly extract peaks from GV collapse signals along the temporal axis. We excluded pre-collapse frames as their inclusion in ${{{\rm{S}}}}$ degraded the quality of BURST images. (In the case of the temporal unmixing algorithm, the insignificance of pre-collapse frames for calculating BURST signals can be mathematically proven, although we omit the proof here for brevity.)

### Doppler imaging sequence

We developed the Doppler imaging sequence by applying the 3D ultrafast Doppler imaging paradigm^23^ to the “Direct” multiplexing combination^29^ of transmit (TX) and receive (RX) banks. In this sequence, for each steering angle, one TX bank transmits a tilted plane wave, and the corresponding RX bank from the direct combinations receives the signals. This process is repeated for each of the four banks. We used six different steering angles: [0°, 0°], [2.5°, –2.5°], [–2.5°, 0°], [2.5°, 2.5°], [0°, –2.5°], and [0°, 2.5°]. Therefore, a total of 4 TX/RX pairs x 6 angles = 24 events were combined through coherently compounding to form a single volume. A power Doppler image was calculated using 200 volume acquisitions with a PRF of 26316 Hz (38 μs between pulse transmissions).

Data transfer and beamforming followed the super-volume methods described in Yu et al.^28^. The RF data from the 200 volume acquisitions were packed into four blocks (or super-volumes), each stored in a different receive frame. Data transfer to the host occurred after each block was obtained. Once data acquisition and storage were complete, beamforming was performed using a separate script specialized to reconstruct IQ data. SVD^61^ was then applied to filter the spatiotemporal IQ data before processing them into a power Doppler image. The voxel size of the reconstructed images was equal to the wavelength for all sides. The FOV of the Doppler images was 9.6 × 9.6 mm.

### GV preparations

As explained in Lakshmanan et al.^63^, after growing the Anabaena cells, GVs were harvested from the floating Anabaena cells. GVs were released from the cells by hypertonic lysis and then purified by using repeated buoyancy-assisted centrifugation and resuspension. GvpC removal was performed by using GV stripping buffer, which contains urea, and by repeated buoyancy-assisted centrifugation and resuspension. The concentration of GVs was measured based on optical density (OD) at 500 nm using a spectrophotometer (Nanodrop 2000c).

### Contrast-to-background ratio (CBR) calculation for in vitro experiments

Phantoms consisted of cylindrical wells filled with GVs embedded in 0.5% agarose, surrounded by a background media made from 0.2% 3 μm Al_2_O_3_ + 1% Agarose. To prepare the GV mixture, GV solutions with an OD of 13 were mixed with 1% agarose in a 1:1 ratio, resulting in a final OD of 6.5. The phantoms (*N* = 6) were submerged in a water bath and imaged with their cylindrical wells aligned parallel to the X axis (Supplementary Fig. 5c–e) and rotated to be parallel to the Y axis (Fig. 2c–e). XY slices were visualized in Fig. 2e and Supplementary Fig. 5e by averaging five adjacent slices. The order of the three ultrasound sequences and the two orientations was permuted for each phantom. We empirically optimized the transmission amplitudes for each sequence (the input voltage for Takoyaki AM = 9.5 V, Sheet pAM = 9 V, 3D xAM = 15 V) to maximize AM signals without GV collapse.

The CBR of the GV phantoms was calculated using the following equation^33^,

5 $${CBR}=\frac{|{\mu }_{{{{\rm{GV}}}}}-{\mu }_{{{{\rm{interspace}}}}}|}{{\sigma }_{{{{\rm{interspace}}}}}}$$

where ${\mu }_{{{{\rm{GV}}}}}$ is the average signal intensity of the two GV wells, ${\mu }_{{{{\rm{interspace}}}}}$ is the average signal intensity of the center interspace between the wells, and ${\sigma }_{{{{\rm{interspace}}}}}$ is the standard deviation of signal intensities in the interspace.

To define the regions corresponding to the GVs and the interspace, we first created a volumetric mask based on the geometry of the phantoms and manually registered it to each volumetric image. The mask was then eroded using a sphere with a radius of 5 voxels (2.5 times the wavelength).

The interspace region was defined as a rectangular box situated between the cylindrical regions of the two GV wells, positioned 7 voxels away from the unshrunk mask of each GV well (Fig. 2f). The cylindrical regions were defined where both wells of the eroded masks exhibited the maximum circular area in a slice. The range along the Z axis was determined by the overlap of two unshrunk wells along the Z axis, further reduced by 7 voxels from the upper end.

When the phantom was aligned parallel to the X axis, two additional steps were taken. First, because of the distortion of images along the Y axis, the distance between the two wells appeared shorter than the actual ground truth. To account for this, the volumetric mask was adjusted based on this shortened distance. For phantoms aligned along the Y axis, the length of the mask was similarly adjusted. Second, since the mask was truncated by the FOV of 3D xAM (the only case of truncation, Supplementary Fig. 5e), we applied the ROIs from 3D xAM images to the corresponding images from the other sequences.

### Image similarity analysis

For each phantom, we obtained transformation matrices between the volumetric masks of the phantom aligned along the X axis (mask X) and the Y axis (mask Y). Specifically, using the Medical Registration Estimator application in MATLAB R2024b (MathWorks), we first manually registered mask Y to the fixed mask X, and then performed automatic deformable registration. After confirming that the registration was successful (structural similarity (SSIM) index ≥ 0.99), we stored the affine transformation matrix and the displacement field produced by these steps.

Next, we applied the transformation matrices to the images of the corresponding phantoms aligned along the Y axis and calculated the multi-scale SSIM (MS-SSIM)^48^ between the transformed images and the images of phantoms aligned along the X axis. The region of interest was set to the bounding box of mask X, and the range along the X axis was further reduced to match the FOV of 3D xAM. The intensity of all images was compressed using a base-10 logarithm before computing their MS-SSIM values.

### Spatial resolution measurements

To measure the spatial resolution of the ultrasound sequences, we used phantoms (*N* = 6 for both axial and lateral resolution measurements) with GVs embedded in 0.5% agarose gel, arranged in a stripe pattern with subwavelength-scale widths. Solidified GV mixtures with the desired OD (OD = 2 for axial resolution, and OD = 3 for lateral resolution) were first prepared by mixing GV solution with 1% agarose in a 1:1 ratio. Next, two different methods were employed to create the stripe patterns for axial and lateral resolution assessments.

For axial resolution (Z axis), phantoms with stripe intervals of 0.2 mm and widths of ~100 μm were created using acoustic patterning^49^, following the method used in Rabut et al.^33^ (Supplementary Fig. 6a). Specifically, the GV mixture was loaded into a custom rectangular mold, and GVs located near the antinodes of 3.7 MHz acoustic standing wave were collapsed by increasing the pressure at the antinodes beyond the collapse threshold. For lateral resolution, we utilized the elevational focusing of a linear array probe (Verasonics, L22-14vX) to generate a sawtooth pattern of GVs with a 1 mm interval. This was achieved by repeatedly moving and collapsing the GVs using plane wave transmissions (Supplementary Fig. 6b). We then selected an XY slice as close to the top (thinnest part) of the sawtooth as possible, ensuring that there were still sufficient GV signals. The depth of the extracted slice was Z = ~7 mm. The voxel sizes of the reconstructed images were set to (X, Y, Z) = (1, 1, 1/8) wavelength for axial resolution measurements and (1/2, 1/2, 1/2) wavelength for lateral resolution. Transmission amplitudes were empirically optimized for each sequence and each phantom type to maximize AM signals while minimizing GV collapse. The optical images of these phantoms were taken with a microscope, transmitting a bright light.

For peak detection, we used the built-in function (findpeaks) in MATLAB 2024b (MathWorks). To reduce dependency between detected peaks and increase their independence, we performed peak detection along lines separated by 6 wavelengths (~0.6 mm) in the lateral directions. Specifically, for axial resolution measurements, the lines along which peaks were detected were spaced 6 wavelengths apart in both the X and Y axes. For lateral resolution measurements along the X (or Y) axis, the lines were spaced 6 wavelengths apart in the Y (or X) axis. Peaks were detected along an axis based on three criteria: (1) peak height must be greater than the average + 5 x standard deviation (std) of baseline signals for axial resolution, or average + 3 x std for lateral resolution; (2) peak prominence must be greater than the average + 5 x std for axial resolution, or average + 2 x std for lateral resolution; and (3) the minimum peak distance must be 0.15 mm for axial resolution, or 0.75 mm for lateral resolution. The baseline was extracted from regions without GV phantoms.

After computing the full-width-half-maximum (FWHM) of the detected peaks from all phantoms, the spatial resolution was calculated as the mean ± std of the FWHM values for each sequence (Fig. 2k and Table 1).

### Imaging GV-expressing brain tumor in mouse brains

GV-expressing U87 cells were prepared by genomic integration of second-generation acoustic reporter genes^10,13,50^. Briefly, HEK293T packaging cells (ATCC, CRL-3216) were maintained in high-glucose DMEM supplemented with GlutaMAX, 1 mM sodium pyruvate (Gibco #10569), 20 mM HEPES (Cytiva #SH30237), 0.1 mM 1X MEM-Non-Essential Amino Acids (Gibco #11140), and 10% fetal bovine serum (Bio-techne #S10350). At 80-90% confluency, cells were transfected with 22 µg pLV (containing the GV gene shown in Fig. 1e of ref.^13^), 22 µg pCMVR8.74 (Addgene #22036), and 4.5 µg pMD2.G (Addgene #12259) plasmids using polyethylenimine (PEI; Polysciences) at a 2.8:1 PEI: DNA mass ratio. Following a 12-h incubation, the transfection media were exchanged for fresh DMEM+ containing 10 mM sodium butyrate for 8 h, after which the media were again replaced with fresh DMEM+ alone. Viral supernatant was collected 48 h later, cleared by centrifugation, and used within 24 h. U87 cells (ATCC, HTB-14) were plated in DMEM+ in 6-well plates 24 h before transduction and infected at 70–90% confluence with 500 µL lentiviral supernatant per well in the presence of 10 µg/mL polybrene (Millipore Sigma #TR1003), followed by spinfection for 50 min at 35 °C and incubation for 16–20 h at 37 °C. After viral media removal, cells were passaged for subsequent experiments. The implantation of these genetically engineered tumor cells and subsequent in vivo ultrasound imaging were conducted on three 5-month-old immunodeficient NSG male mice (Jackson Laboratory; denoted as mouse 1: Figs. 3 and 5b, mouse 2; Supplementary Fig. 7, and mouse 3; Supplementary Fig. 9a). All cell lines were authenticated via short tandem repeat profiling and tested negative for mycoplasma contamination.

1 × 10^5^ genetically engineered U87 cells were implanted at coordinates anterior-posterior (AP) –2 mm, medial-lateral (ML) + 1.5 mm, and dorsal-ventral (DV) –3.5 mm. After allowing the tumor cells to grow for 6 days, we injected 150 μl of doxycycline through intraperitoneal (IP) injection for two consecutive days to induce the GV expression in the tumors. Tumor growth did not exceed the IACUC-approved limits, as assessed by associated clinical signs and humane endpoints. One day after the final doxycycline injection, the mice were anesthetized with 2-3% isoflurane, and a portion of the skull was removed to facilitate ultrasound imaging. After applying ultrasound gel, we initially imaged the mouse brain using a linear array probe (Verasonics, L22-14vX; center frequency 15.625 MHz) by taking xAM (angle = 19.5°, aperture size = 6.5 mm, PRF = 2 kHz)^32^ images of coronal slices with a 0.2 mm interval (except for mouse 3). The linear array probe was mounted on a stereotaxic instrument (Kopf Instruments) and translated by manually rotating a control knob, while its position was monitored using a digital display console. Next, the mouse brain was imaged with a matrix array probe using Takoyaki B-mode, AM and finally, BURST sequences. After collapsing the GVs, power Doppler images were acquired using the matrix array at the same location (except for mouse 2).

We used Napari^64^ for visualizing the volume images. The FOV of the Doppler images was 9.6 × 9.6 mm, but the volume was cropped to match the FOV of Takoyaki AM and BURST. The dimensions of the volume image were (X, Y, Z) = 7.5 × 8.4 × 5.9 mm, with a depth range from 3.4 mm to 9.3 mm (Fig. 3b). For the anterior and lateral views, the volume images were half-cropped to exclude the skull side. The stacked 2D xAM (st-xAM) images were resized using bicubic interpolation from a voxel size of 50 × 200 × 1 *μ*m to 50 × 50 × 50 *μ*m so that they can be visualized in Napari with a reduced number of voxels. The volume size of the st-xAM image was 6.4 × 2.6 × 9 mm (Fig. 3e). We then manually registered the st-xAM image based on known probe locations relative to the bregma, patterns of GV signals and characteristic artifacts.

For CBR calculations, we drew a bounding box inscribed within the tumor to define the GV signal region for both Takoyaki AM and st-xAM images. The background region was defined as a bounding box of the same size, located at the contralateral counterpart of the tumor.

### Real-time monitoring of GV injections in the brain ventricles

The in vivo real-time monitoring experiment was performed on two 6-month-old C37bl/6 male mice (Jackson Laboratory, denoted as mouse 4: Figs. 4 and 5c, and mouse 5: Supplementary Figs. 8 and 9b). The mice were anesthetized with 2–3% isoflurane, and a portion of the skull was removed for imaging. A 10 µL microliter syringe (Hamilton) was tilted at 45 degrees and targeted the right lateral ventricle of the mouse brain (AP -0.5 mm, ML + 1 mm, DV -2 mm). A total of 4.5 $\mu$L of GV solution (OD 14, equivalent to 1.6 nM^63^) was injected over 60 s at a rate of 75 nL s^–1^ using a micro syringe pump (World Precision Instrument), starting between the 11th and 12th frames. After applying ultrasound gel, a matrix probe was tilted at less than 10 degrees and placed without touching the micro-syringe.

Real-time imaging of the mouse brain was performed through the skull-removed window using the Takoyaki AM sequence, capturing data up to the 150th frame for mouse 4 (300th frame for mouse 5), which took approximately 10 mins (20 mins). Image reconstruction and necessary computations were performed using an Intel Xeon Gold 6136 CPU. The actual real-time visualization of live images was handled using Verasonics Vantage's built-in functions, which displayed X, Y, and Z slices and volume images. Image reconstruction/visualization (~2.8 s/frame) and storage (~1 s/frame) were done after acquiring data for each frame, yielding a frame rate of 3.8 s/frame. Power Doppler images were obtained before GV injection at the same location. After completing real-time monitoring, a BURST image was acquired using the same script as for the real-time monitoring session, but with a high-pressure setting (Voltage ≥ 28 V).

For analysis, the Volume Segmenter App in MATLAB 2024b (MathWorks) was used to manually label the lateral ventricles and the 3rd ventricles. Regions affected by artifacts from the needle were excluded from the labels. Signal density was calculated by summing all signals within a labeled volume and dividing by the volume. 3D visualization of the stored images was done using Napari. The dimensions of the cropped Takoyaki AM image were (X, Y, Z) = 7.5 × 4.2 × 6.3 mm, with a depth range from 3.3 mm to 9.6 mm (Fig. 4d).

### Hydrophone measurements

Hydrophone measurements were performed in a water tank filled with degassed water, maintained using a water conditioner (Onda). The matrix probe was mounted on a motorized positioning stage (Velmex XSlide). A needle hydrophone (Onda HNR-0500) was submerged and fixed at the bottom of the tank, with its tip oriented perpendicularly toward the surface of the matrix probe. The hydrophone and Vantage 256 were connected to a digital oscilloscope (Keysight DSOX2004A) to measure acoustic pressure upon receiving trigger signals from the Vantage 256. Translating the probe across the field of interest, acoustic pressure was measured at each grid point. After averaging 256 received signals on the oscilloscope at each position, the averaged signal was sent to a connected computer, and the peak-to-peak pressure was calculated. For more accurate pressure measurements at the focus of the Takoyaki sequence (1^st^ TX aperture, Supplementary Fig. 2a) within the Z = 7 mm plane, a fiber optic hydrophone system (Precision Acoustics) was used instead of the needle hydrophone. The pressure of plane waves was also measured at the same location using the fiber optic hydrophone (Supplementary Fig. 4b).

### Statistics and reproducibility

For the in vitro experiments, the sample size was decided based on preliminary studies to cover all six permutations of the three image sequences and to provide adequate statistical power. These sequence orders were assigned across randomly selected phantoms. For the in vivo experiments, the objective was to demonstrate the effectiveness of our imaging methods in living animals as a proof of concept. Accordingly, two or three animals were used for each experiment. Images from biological replicates not shown in the main figures are provided in the Supplementary Figs. Animals were randomly chosen from their cages. CBRs and MS-SSIM values were compared between each pair of imaging sequences using a two-sided paired samples t-test. Differences in spatial resolution among sequences were assessed using the two-sided Mann-Whitney U test. Statistical analyses were performed using MATLAB 2024b. No data were excluded from the analyses. Sex was not considered as a factor in the study design or analysis because the study was primarily intended to evaluate imaging performance. Blinding was not performed because it was not expected to affect the outcomes of this imaging study.

### Reporting summary

Further information on research design is available in the Nature Portfolio Reporting Summary linked to this article.

## Supplementary information

Supplementary Information

Reporting Summary

Description of additional supplementary files

Supplementary Movie 1

Supplementary Movie 2

Supplementary Movie 3

Supplementary Movie 4

Transparent Peer Review file

## Source data

Source Data

## Data Availability

Primary image data have been deposited in the CaltechDATA repository at 10.22002/knnyt-x4762, available at https://data.caltech.edu/records/knnyt-x4762. Source data are provided with this paper.

## Code availability

The ultrasound sequence scripts and data analysis codes used to generate key figures and results are available on GitHub (https://github.com/shapiro-lab/Takoyaki-imaging)^65^.

## References

[1] Huang, Q. & Zeng, Z. A review on real-time 3D ultrasound imaging technology. *BioMed Res. Int.* **2017**, 6027029 (2017). 28459067 10.1155/2017/6027029 PMC5385255

[2] Yusefi, H. & Helfield, B. Ultrasound contrast imaging: fundamentals and emerging technology. *Front. Phys*. **10**, 791145 (2022).

[3] Frinking, P., Segers, T., Luan, Y. & Tranquart, F. Three decades of ultrasound contrast agents: a review of the past, present and future improvements. *Ultrasound Med. Biol.* **46**, 892–908 (2020). 31941587 10.1016/j.ultrasmedbio.2019.12.008

[4] de Leon, A. et al. Contrast-enhanced ultrasound imaging by nature-inspired ultrastable echogenic nanobubbles. *Nanoscale* **11**, 15647–15658 (2019). 31408083 10.1039/c9nr04828f PMC6716144

[5] Sheeran, P. S. & Dayton, P. A. Phase-change contrast agents for imaging and therapy. *Curr. Pharm. Des.* **18**, 2152–2165 (2012). 22352770 10.2174/138161212800099883 PMC5045864

[6] Shapiro, M. G. et al. Biogenic gas nanostructures as ultrasonic molecular reporters. *Nat. Nanotechnol.* **9**, 311–316 (2014). 24633522 10.1038/nnano.2014.32 PMC4023545

[7] Walsby, A. E. Gas vesicles. *Microbiol. Rev.* **58**, 94–144 (1994). 8177173 10.1128/mr.58.1.94-144.1994 PMC372955

[8] Bourdeau, R. W. et al. Acoustic reporter genes for noninvasive imaging of microorganisms in mammalian hosts. *Nature* **553**, 86–90 (2018). 29300010 10.1038/nature25021 PMC5920530

[9] Farhadi, A., Ho, G. H., Sawyer, D. P., Bourdeau, R. W. & Shapiro, M. G. Ultrasound imaging of gene expression in mammalian cells. *Science* **365**, 1469–1475 (2019). 31604277 10.1126/science.aax4804 PMC6860372

[10] Hurt, R. C. et al. Genomically mined acoustic reporter genes for real-time in vivo monitoring of tumors and tumor-homing bacteria. *Nat. Biotechnol.* **41**, 919–931 (2023). 36593411 10.1038/s41587-022-01581-y PMC10344784

[11] Buss, M. T., Zhu, L., Kwon, J. H., Tabor, J. J. & Shapiro, M. G. Probiotic acoustic biosensors for noninvasive imaging of gut inflammation. *Nat. Commun.* **16**, 7931 (2025). 40855083 10.1038/s41467-025-62569-1 PMC12379287

[12] Nyström, N. N. et al. Multiplexed ultrasound imaging of gene expression. *Nat. Methods* **22**, 2594–2600 (2025). 41254367 10.1038/s41592-025-02825-w PMC12695634

[13] Shivaei, S. et al. Non-invasive imaging of cell-based therapies using acoustic reporter genes. Preprint at 10.1101/2024.11.01.621111 (2024).

[14] Jin, Z. et al. Ultrasonic reporters of calcium for deep tissue imaging of cellular signals. Preprint at 10.1101/2023.11.09.566364 (2023).

[15] Lakshmanan, A. et al. Acoustic biosensors for ultrasound imaging of enzyme activity. *Nat. Chem. Biol.* **16**, 988–996 (2020). 32661379 10.1038/s41589-020-0591-0 PMC7713704

[16] Le Floc’h, J. et al. In vivo biodistribution of radiolabeled acoustic protein nanostructures. *Mol. Imaging Biol.* **20**, 230–239 (2018). 28956265 10.1007/s11307-017-1122-6 PMC6110388

[17] Wang, G. et al. Surface-modified GVs as nanosized contrast agents for molecular ultrasound imaging of tumor. *Biomaterials* **236**, 119803 (2020). 32028170 10.1016/j.biomaterials.2020.119803

[18] Ling, B. et al. Biomolecular ultrasound imaging of phagolysosomal function. *ACS Nano* **14**, 12210–12221 (2020). 32902951 10.1021/acsnano.0c05912 PMC7685203

[19] Wang, Y. et al. Modification of PEG reduces the immunogenicity of biosynthetic gas vesicles. *Front. Bioeng. Biotechnol.* **11**, 1128268 (2023). 36949883 10.3389/fbioe.2023.1128268 PMC10025544

[20] Ling, B. et al. Gas vesicle–blood interactions enhance ultrasound imaging contrast. *Nano Lett* **23**, 10748–10757 (2023). 37983479 10.1021/acs.nanolett.3c02780 PMC10722532

[21] Ling, B. et al. Truly tiny acoustic biomolecules for ultrasound imaging and therapy. *Adv. Mater.* **36**, 2307106 (2024). 10.1002/adma.202307106 PMC11602542 38409678

[22] Provost, J. et al. 3D ultrafast ultrasound imaging in vivo. *Phys. Med. Biol.* **59**, L1 (2014). 25207828 10.1088/0031-9155/59/19/L1 PMC4820600

[23] Provost, J. et al. 3-D ultrafast Doppler imaging applied to the noninvasive mapping of blood vessels in Vivo. *IEEE Trans. Ultrason. Ferroelectr. Freq. Control* **62**, 1467–1472 (2015). 26276956 10.1109/TUFFC.2015.007032 PMC4993233

[24] Roux, E. et al. Experimental 3-D ultrasound imaging with 2-D sparse arrays using focused and diverging waves. *Sci. Rep.* **8**, 9108 (2018). 29904182 10.1038/s41598-018-27490-2 PMC6002520

[25] Heiles, B. et al. Ultrafast 3D ultrasound localization microscopy using a 32 \times 32 matrix array. *IEEE Trans. Med. Imaging* **38**, 2005–2015 (2019). 30946662 10.1109/TMI.2018.2890358

[26] Rabut, C. et al. 4D functional ultrasound imaging of whole-brain activity in rodents. *Nat. Methods* **16**, 994 (2019). 31548704 10.1038/s41592-019-0572-y PMC6774790

[27] Sauvage, J. et al. 4D functional imaging of the rat brain using a large aperture row-column array. *IEEE Trans. Med. Imaging* **39**, 1884–1893 (2020). 31841403 10.1109/TMI.2019.2959833

[28] Yu, J., Yoon, H., Khalifa, Y. M. & Emelianov, S. Y. Design of a volumetric imaging sequence using a Vantage-256 ultrasound research platform multiplexed with a 1024-element fully-sampled matrix array. *IEEE Trans. Ultrason. Ferroelectr. Freq. Control* **67**, 248–257 (2020). 31545718 10.1109/TUFFC.2019.2942557 PMC7008949

[29] Chavignon, A. et al. 3D transcranial ultrasound localization microscopy in the rat brain with a multiplexed matrix probe. *IEEE Trans. Biomed. Eng.* **69**, 2132–2142 (2022). 34932470 10.1109/TBME.2021.3137265

[30] Schmidt, E. L. et al. Near-infrared II fluorescence imaging. *Nat. Rev. Methods Primer* **4**, 1–22 (2024).

[31] Maresca, D. et al. Nonlinear ultrasound imaging of nanoscale acoustic biomolecules. *Appl. Phys. Lett.* **110**, 073704 (2017). 28289314 10.1063/1.4976105 PMC5315666

[32] Maresca, D., Sawyer, D. P., Renaud, G., Lee-Gosselin, A. & Shapiro, M. G. Nonlinear X-wave ultrasound imaging of acoustic biomolecules. *Phys. Rev. X* **8**, 041002 (2018). 34040818 10.1103/physrevx.8.041002 PMC8147876

[33] Rabut, C. et al. Ultrafast amplitude modulation for molecular and hemodynamic ultrasound imaging. *Appl. Phys. Lett.* **118**, 244102 (2021). 34176950 10.1063/5.0050807 PMC8205510

[34] Sawyer, D. P. et al. Ultrasensitive ultrasound imaging of gene expression with signal unmixing. *Nat. Methods* **18**, 945–952 (2021). 34354290 10.1038/s41592-021-01229-w PMC8363212

[35] Cherin, E. et al. Acoustic behavior of halobacterium salinarum gas vesicles in the high-frequency range: experiments and modeling. *Ultrasound Med. Biol.* **43**, 1016–1030 (2017). 28258771 10.1016/j.ultrasmedbio.2016.12.020 PMC5385285

[36] Zhang, S. et al. The vibration behavior of sub-micrometer gas vesicles in response to acoustic excitation determined via laser Doppler vibrometry. *Adv. Funct. Mater.* **30**, 2000239 (2020).

[37] Rasmussen, M. F., Christiansen, T. L., Thomsen, E. V. & Jensen, J. A. 3-D imaging using row-column-addressed arrays with integrated apodization—part I: apodization design and line element beamforming. *IEEE Trans. Ultrason. Ferroelectr. Freq. Control* **62**, 947–958 (2015). 25974918 10.1109/TUFFC.2014.006531

[38] Christiansen, T. L. et al. 3-D imaging using row–column-addressed arrays with integrated apodization— part ii: transducer fabrication and experimental results. *IEEE Trans. Ultrason. Ferroelectr. Freq. Control* **62**, 959–971 (2015). 25974919 10.1109/TUFFC.2014.006819

[39] Bouzari, H. et al. Imaging performance for two-row column arrays. *IEEE Trans. Ultrason. Ferroelectr. Freq. Control* **66**, 1209–1221 (2019). 31056493 10.1109/TUFFC.2019.2914348

[40] Heiles, B. et al. Nonlinear sound-sheet microscopy: imaging opaque organs at the capillary and cellular scale. *Science* **388**, eads1325 (2025). 40179181 10.1126/science.ads1325 PMC12661648

[41] Bernal, M., Cunitz, B., Rohrbach, D. & Daigle, R. High-frame-rate volume imaging using sparse-random-aperture compounding. *Phys. Med. Biol.* **65**, 175002 (2020). 32413874 10.1088/1361-6560/ab9372

[42] Xing, P. et al. 3D ultrasound localization microscopy of the nonhuman primate brain. *eBioMedicine* **111**, 105457 (2025). 39708427 10.1016/j.ebiom.2024.105457 PMC11730257

[43] Mallart, R. & Fink, M. Improved imaging rate through simultaneous transmission of several ultrasound beams. In: *New Developments in Ultrasonic Transducers and Transducer Systems* vol. 1733 120–130 (SPIE, 1992).

[44] Denarie, B., Bjåstad, T. & Torp, H. Multi-line transmission in 3-D with reduced crosstalk artifacts: a proof of concept study. *IEEE Trans. Ultrason. Ferroelectr. Freq. Control* **60**, 1708–1718 (2013). 25004541 10.1109/TUFFC.2013.2752

[45] Ortega, A. et al. A Comparison of the performance of different multiline transmit setups for fast volumetric cardiac ultrasound. *IEEE Trans. Ultrason. Ferroelectr. Freq. Control* **63**, 2082–2091 (2016). 27705857 10.1109/TUFFC.2016.2614652

[46] Badescu, E., Bujoreanu, D., Petrusca, L., Friboulet, D. & Liebgott, H. Multi-line transmission for 3d ultrasound imaging: an experimental study. In *Proc.**2017 IEEE International Ultrasonic Symposium (IUS)* (IEEE, 2017).

[47] McCall, J. R., Chavignon, A., Couture, O., Dayton, P. A. & Pinton, G. F. Element position calibration for matrix array transducers with multiple disjoint piezoelectric panels. *Ultrason. Imaging* **46**, 139–150 (2024). 38334055 10.1177/01617346241227900

[48] Wang, Z., Simoncelli, E. P. & Bovik, A. C. Multiscale structural similarity for image quality assessment. In *Proc.**Thrity-Seventh Asilomar Conference on Signals, Systems & Computers, 2003* (IEEE, 2003).

[49] Wu, D. et al. Biomolecular actuators for genetically selective acoustic manipulation of cells. *Sci. Adv.* **9**, eadd9186 (2023). 36812320 10.1126/sciadv.add9186 PMC9946353

[50] Rabut, C., Shivaei, S., Heiles, B. & Shapiro, M. G. Trimodal brain-wide ultrasound imaging of brain-tumor interaction. Preprint at 10.1101/2025.10.29.685462 (2025).

[51] Vert, M. et al. Transcranial brain-wide functional ultrasound and ultrasound localization microscopy in mice using multi-array probes. *Sci. Rep.* **15**, 12042 (2025). 40199928 10.1038/s41598-025-96647-7 PMC11978944

[52] Xie, L. et al. Sleep drives metabolite clearance from the adult brain. *Science* **342**, 373–377 (2013). 24136970 10.1126/science.1241224 PMC3880190

[53] Kelley, D. H. Brain cerebrospinal fluid flow. *Phys. Rev. Fluids* **6**, 070501 (2021). 34671708 10.1103/physrevfluids.6.070501 PMC8525801

[54] Kelley, D. H. & Thomas, J. H. Cerebrospinal fluid flow. *Annu. Rev. Fluid Mech.* **55**, 237–264 (2023). 39691763 10.1146/annurev-fluid-120720-011638 PMC11651633

[55] Hughes, M. P. et al. AAV9 intracerebroventricular gene therapy improves lifespan, locomotor function and pathology in a mouse model of Niemann–Pick type C1 disease. *Hum. Mol. Genet.* **27**, 3079–3098 (2018). 29878115 10.1093/hmg/ddy212 PMC6097154

[56] Taghian, T. et al. A safe and reliable technique for CNS delivery of AAV vectors in the cisterna magna. *Mol. Ther.* **28**, 411–421 (2020). 31813800 10.1016/j.ymthe.2019.11.012 PMC7002897

[57] Rabut, C. et al. Acoustic tumor paint for real-time imaging, surgical guidance and recurrence monitoring of brain tumors with ultrasound. Preprint at 10.1101/2024.12.22.629782 (2024).

[58] Shivaei, S. et al. Ultrasound imaging of in situ transcriptional activity in opaque tissue. Preprint at 10.1101/2025.07.06.663365 (2025).

[59] Hurt, R. C. et al. Plasmid from article—genomically mined acoustic reporter genes for real-time in vivo monitoring of tumors and tumor-homing bacteria. *Addgene* https://www.addgene.org/browse/article/28229434/. 10.1038/s41587-022-01581-y PMC10344784 36593411

[60] Bureau, F. et al. Three-dimensional ultrasound matrix imaging. *Nat. Commun.* **14**, 6793 (2023). 37880210 10.1038/s41467-023-42338-8 PMC10600255

[61] Demené, C. et al. Spatiotemporal clutter filtering of ultrafast ultrasound data highly increases Doppler and ultrasound sensitivity. *IEEE Trans. Med. Imaging* **34**, 2271–2285 (2015). 25955583 10.1109/TMI.2015.2428634

[62] Eckart, C. & Young, G. The approximation of one matrix by another of lower rank. *Psychometrika* **1**, 211–218 (1936).

[63] Lakshmanan, A. et al. Preparation of biogenic gas vesicle nanostructures for use as contrast agents for ultrasound and MRI. *Nat. Protoc.* **12**, 2050–2080 (2017). 28880278 10.1038/nprot.2017.081 PMC6185898

[64] Ahlers, J. et al. Napari: a multi-dimensional image viewer for Python. Zenodo 10.5281/zenodo.8115575 (2023).

[65] Lee, S. Shapiro-Lab/Takoyaki-imaging: Takoyaki imaging. Zenodo 10.5281/zenodo.19124225 (2026).

[66] Wang, Q. et al. The Allen Mouse Brain Common Coordinate Framework: a 3D reference atlas. *Cell* **181**, 936–953.e20 (2020). 32386544 10.1016/j.cell.2020.04.007 PMC8152789

