# Supplementary material for: Real-time volumetric imaging of cells and molecules in deep tissues with Takoyaki ultrasound
Source: Nat Commun. 2026 May 21;17:7281. doi: 10.1038/s41467-026-72961-0 (PMC13402839; doi:10.1038/s41467-026-72961-0)
Supplement: Supplementary file 1 — Supplementary Information [file 41467_2026_72961_MOESM1_ESM.pdf]

**Supplementary Information**

# Real-time volumetric imaging of cells and molecules in deep tissues with Takoyaki ultrasound

Sunho Lee[1], Di Wu[2], Dina Malounda[2], Claire Rabut[2*], Mikhail G. Shapiro[1,2,3*]

[1]Andrew and Peggy Cherng Department of Medical Engineering, California Institute of Technology, Pasadena, CA, USA
[2]Division of Chemistry and Chemical Engineering, California Institute of Technology, Pasadena, CA, USA
[3]Howard Hughes Medical Institute, Pasadena, CA, USA

[*] Correspondence should be addressed to:
CR (crabut@caltech.edu), MGS (mikhail@caltech.edu)

## Contents:

Supplementary Figures 1-9.

Supplementary Notes 1-2.

## Supplementary Figures

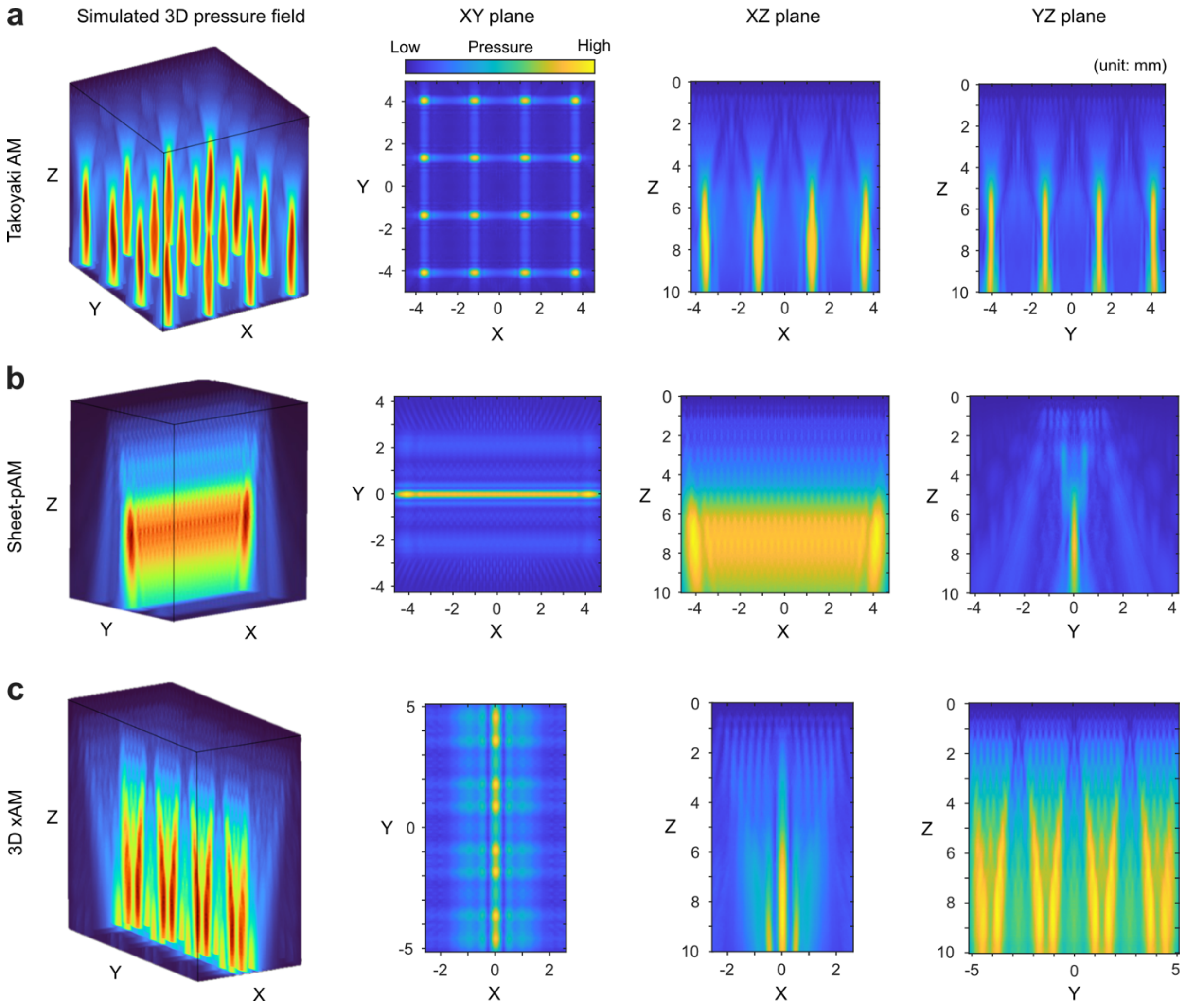


**Supplementary Figure 1: Simulated pressure fields**. Pressure fields of Takoyaki (**a**), Sheet-pAM (**b**), and 3D xAM (**c**) simulated with TXPD in Verasonics Vantage software, based on the delays for full-amplitude shown in **Fig. 2b**. XY planes are extracted from Z = 7 mm. XZ planes of Takoyaki, Sheet-pAM, and 3D xAM pressure fields correspond to Y = 1.33 mm, Y = 0, and Y = 0.99 mm, respectively. YZ planes of Takoyaki, Sheet-pAM, and 3D xAM pressure fields correspond to X = 1.18 mm, X = 0, and X = 0, respectively.

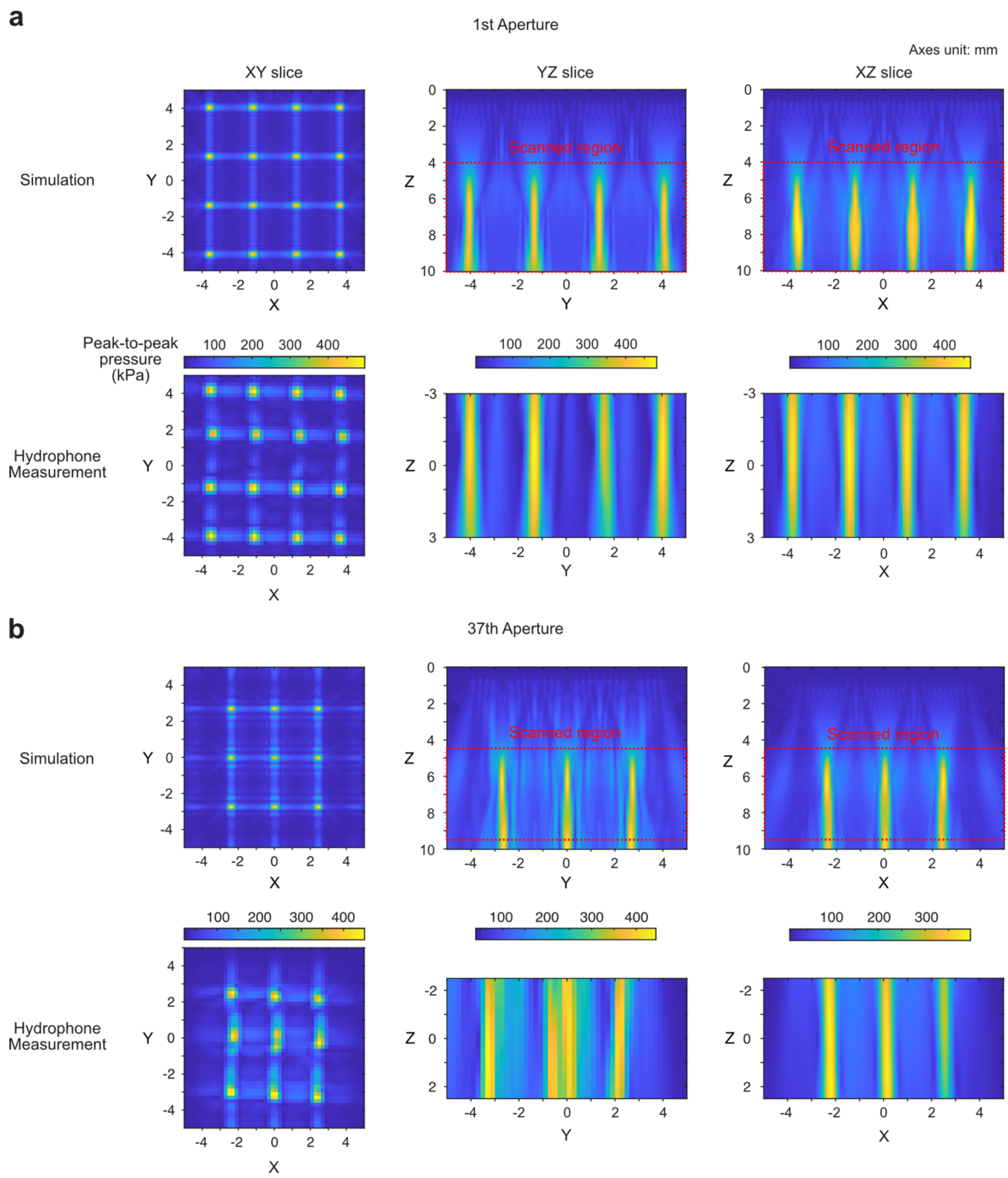


**Supplementary Figure 2**: **Measured pressured fields of Takoyaki sequence**. Pressure fields of the Takoyaki sequence (focus = 7 mm) measured with a needle hydrophone. Their simulated pressure fields (ground truths) are shown in the top row of each panel. The coordinates of the hydrophone are centered at (0, 0, 7) mm in the simulation coordinate system. Red dotted boxes represent the regions scanned by the hydrophone. **a**. The first transmit aperture generates a (4 x 4) mesh of focused ultrasound. The XY, YZ, and XZ slices correspond to positions Z = 7 mm, X = 1.18 mm, and Y = 1.33 mm, respectively. While the focal points remain well-defined, deviations in their locations imply misalignment among the transducer element banks. This type of misalignment – where between-bank misalignment is more pronounced than with-bank misalignment – was also noted or assumed in previous element position calibration study for similar matrix array probe[1]. **b**. The 37$^{th}$ transmit aperture emits a (3 x 3) mesh of focused ultrasound at the center of the FOV, with all focal points located within gap areas. The XY, YZ, and XZ slices correspond to positions Z = 7 mm, X = 0 mm, and Y = 0 mm, respectively. The pressure field at the Y = 0 mm plane is disrupted, likely due to the element bank misalignment. Combined with inherent gaps in the matrix array, which can hinder uniform pressure field application along the Y axis, these bank misalignments appear to cause the weakening of signals around Y = 0 mm.

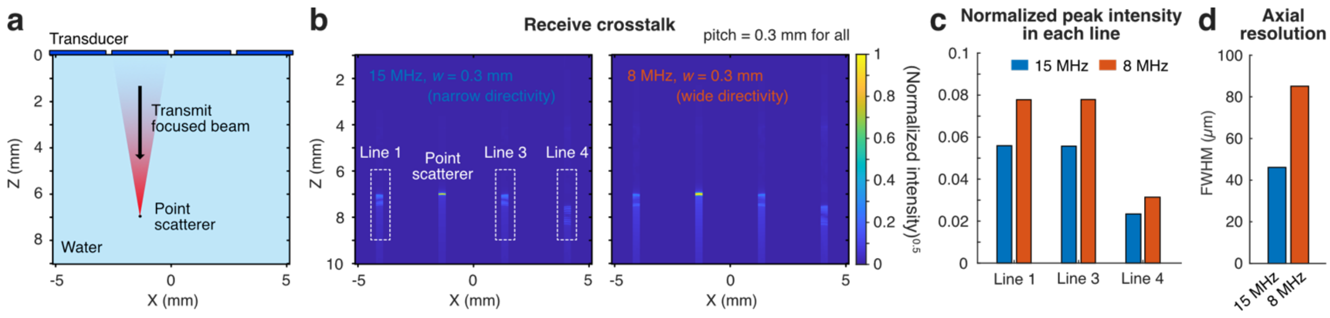


**Supplementary Figure 3: Receive crosstalk among multiple focused beams depending on element directivity. a**. 2D simulation of receive crosstalk performed using k-wave. The medium is modeled as water with homogeneous density and sound speed. The element positions (element width $w$ = 0.3 mm) match those in this paper. Four ray lines are reconstructed based on the delay function of four focused beams (8 elements for each) after a point scatterer (air bubble) placed on the second ray line is insonified with a focused beam from the second bank. Grid spacing = 3.08 μm. cfl = 0.5. **b**. Simulated images revealing receive crosstalk at transmit frequencies of 15 MHz (left) and 8 MHz (right). The pressure at the point scatterer is matched across simulations. Beamforming at each pixel uses the identical set of received RF signals (sensitivity cutoff = 0.6 for the case of 15 MHz). Reconstructed images are normalized to the maximum intensity and power law compressed (exponent = 0.5). Pixel size = 12.5 μm. **c**. Quantification of receive crosstalk, expressed as the peak intensity measured along each line. **d**. Axial resolution (full-width-half-maximum; FWHM) measured at the point scatterer. Source data are provided as a Source Data file.

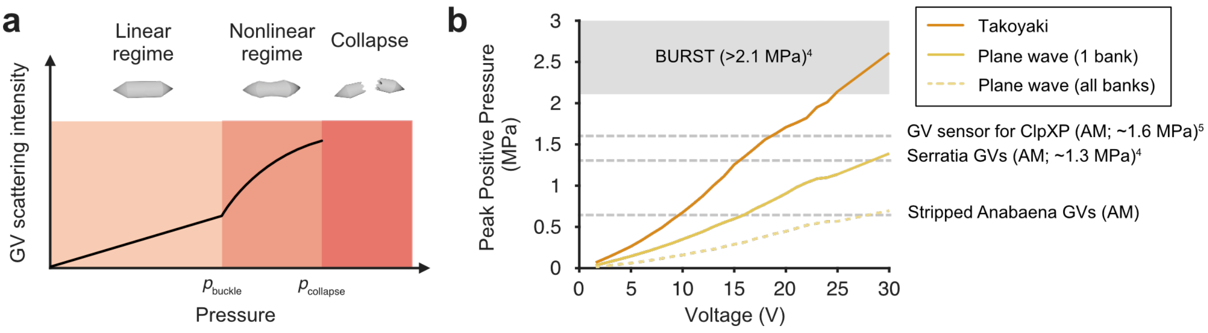


**Supplementary Figure 4: Pressure ranges of Takoyaki and plane-wave-based sequences**. **a**. Illustration of different GV behaviors as a function of ultrasound pressure. Below the buckling threshold ($p_{\text{buckle}}$), GVs exhibit linear scattering. Between $p_{\text{buckle}}$ and the collapse threshold ($p_{\text{collapse}}$), GVs begin to buckle, producing nonlinear responses suitable for AM imaging. Above $p_{\text{collapse}}$, GVs collapse and generate strong, transient signals that can be isolated from the background using the BURST paradigm. **b**. Peak positive pressure of the Takoyaki sequence and plane waves emitted using one bank[2] and all banks[3], plotted against the input voltage of the MMA transducer. With the Verasonics Vantage 256, the acoustic output decreases when more banks are used due to multiple hardware issues. Gray dashed lines and shaded area mark the optimal pressure levels for AM and BURST imaging, respectively. For certain types of GVs (e.g. Serratia GVs[4] and ClpXP GV sensor[5]), the pressure produced by plane waves is not enough to reach the optimum pressure for AM imaging or requires nearly the maximum voltage (30V) regarding the safety of matrix probes. This is more challenging for *in vivo* applications, where higher pressure is needed to compensate for tissue attenuation compared to *in vitro* settings. The pressures are measured at one of the focal points (Z = 7 mm) of the Takoyaki transmission shown in **Fig. S2a**, using a fiber optic hydrophone. Pressure values for 25 – 30 V are extrapolated using linear fitting. Source data are provided as a Source Data file.

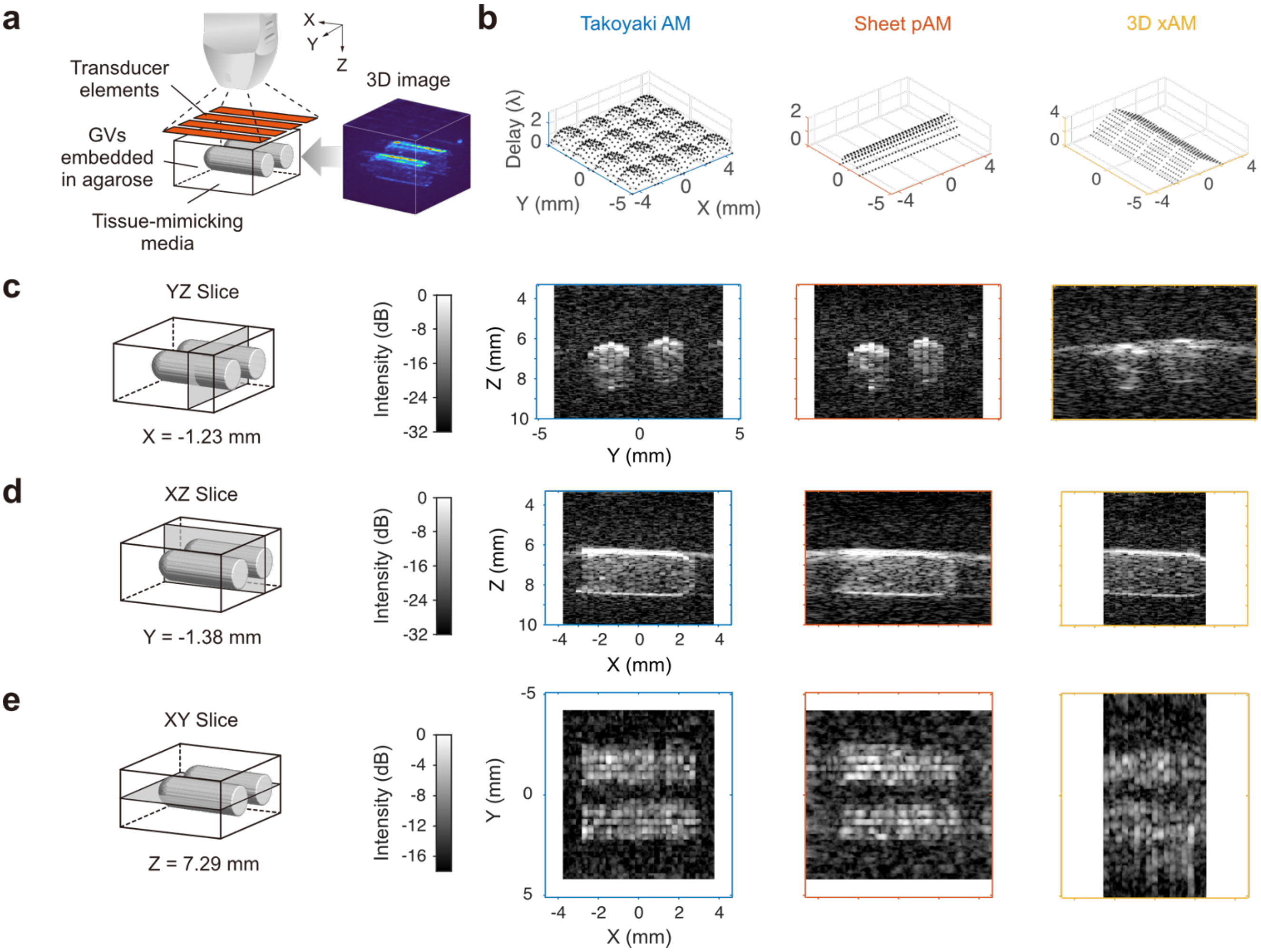


**Supplementary Figure 5: AM images of 90-degree rotated phantoms**. **a**. Experiment setup for imaging tissue-mimicking GV phantoms. The orange parallelograms represent the four banks of transducer elements. The axis of the wells was aligned with the X axis, and the phantom location was adjusted so that its center was situated at Z = 7 mm. The 3D image of the phantom (OD = 6.5) acquired with Takoyaki AM is shown on the right. **b**. Representative delay laws of each sequence. **c-e**. Sliced phantom images acquired using different ultrasound sequences. For XY slices, five slices are averaged. The outer colored lines surrounding the sliced images indicate the maximum FOV. The color scale of the images is adjusted to match background levels across sequences.

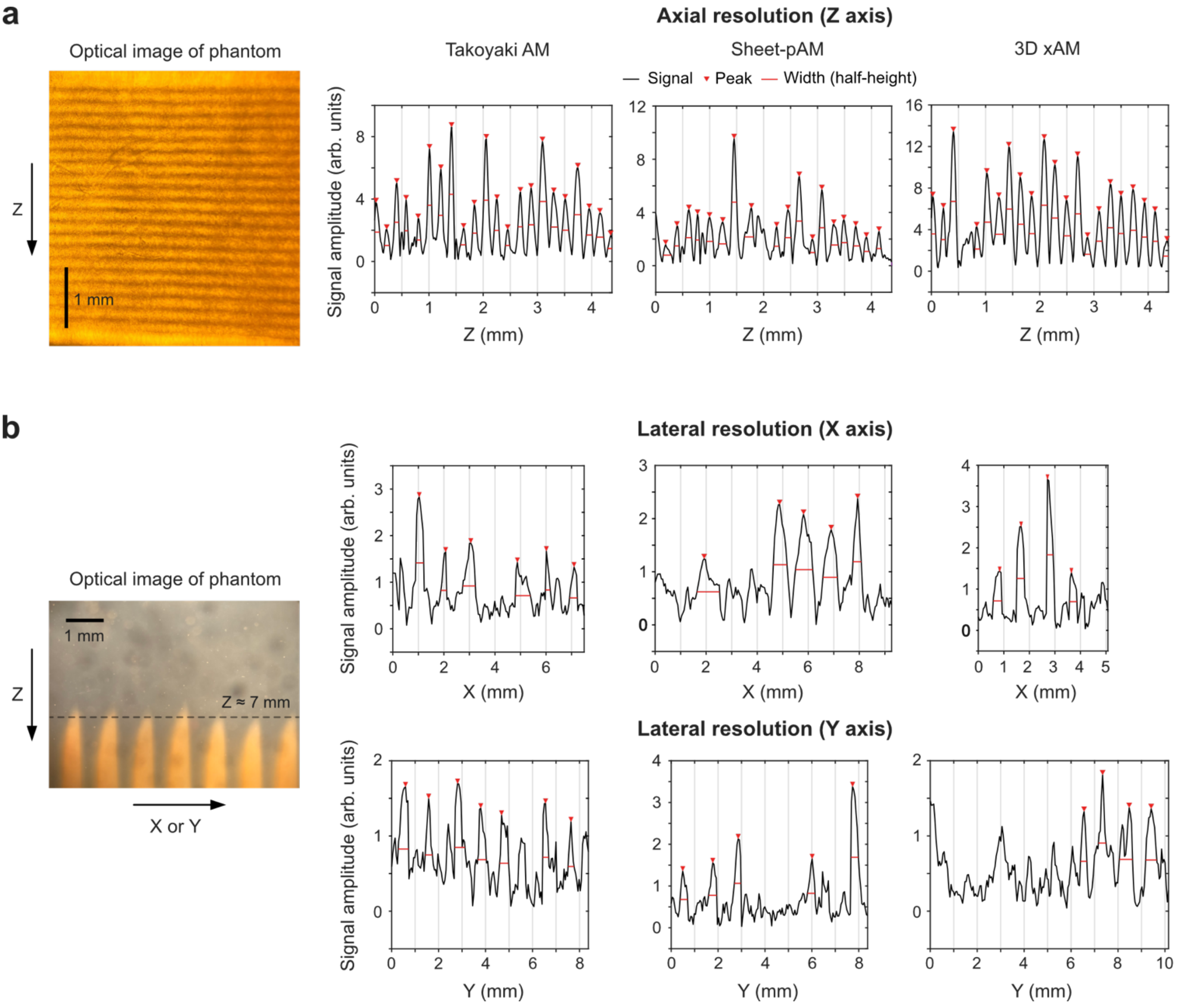


**Supplementary Figure 6: Spatial resolution measurements using phantoms with GV stripes**. Detected peaks and their full-width-half-maximum (FWHM) are marked with red triangles and red lines, respectively. **a**. Axial (Z axis) resolution measurements. Phantoms have GV stripes with an interval of 0.2 mm. For each sequence, an example of signals along the Z axis is shown. **b**. Lateral (X and Y axes) resolution measurements. Phantoms have a saw-tooth pattern of GVs with the interval of 1 mm, and we chose the plane of Z = ~7 mm (sharp regions of the saw-tooth) for FWHM analysis. The top and bottom rows correspond to the X and Y directions, respectively. Source data are provided as a Source Data file.

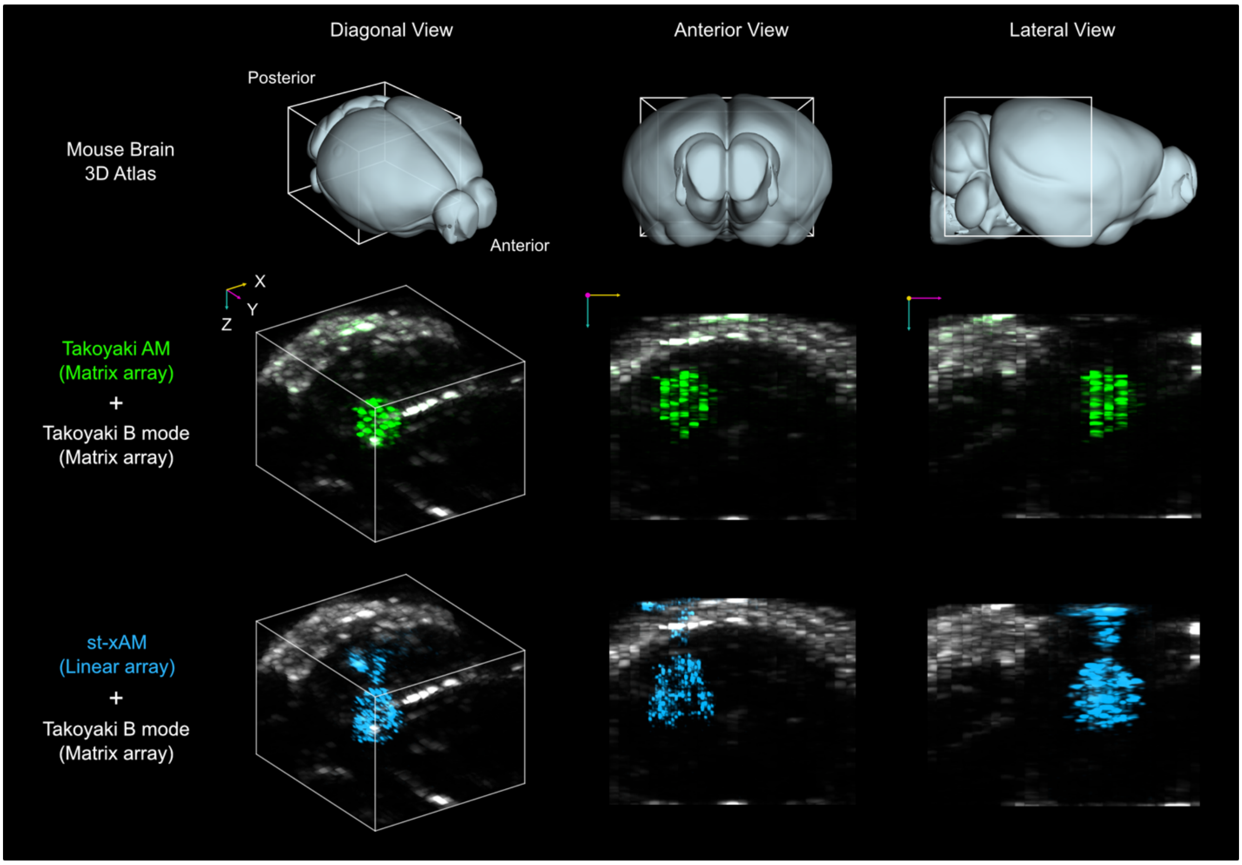


**Supplementary Figure 7: Additional example of imaging genetically labeled tumor in mouse brain with the Takoyaki AM and st-xAM**. Both images (Takoyaki AM – green; st-xAM – blue) are overlaid with Takoyaki B-mode (white). The size of the left column's volume images is (X, Y, Z) = 7.5 x 8.4 x 6.3 mm, with a depth range from 3.4 mm to 9.7 mm. The white box on the brain atlas indicates the imaging regions. Colored arrows represent the axes, with lengths of 1 mm along their corresponding directions. CBRs for Takoyaki AM and st-xAM were 11.27 and 10.33 dB, respectively.

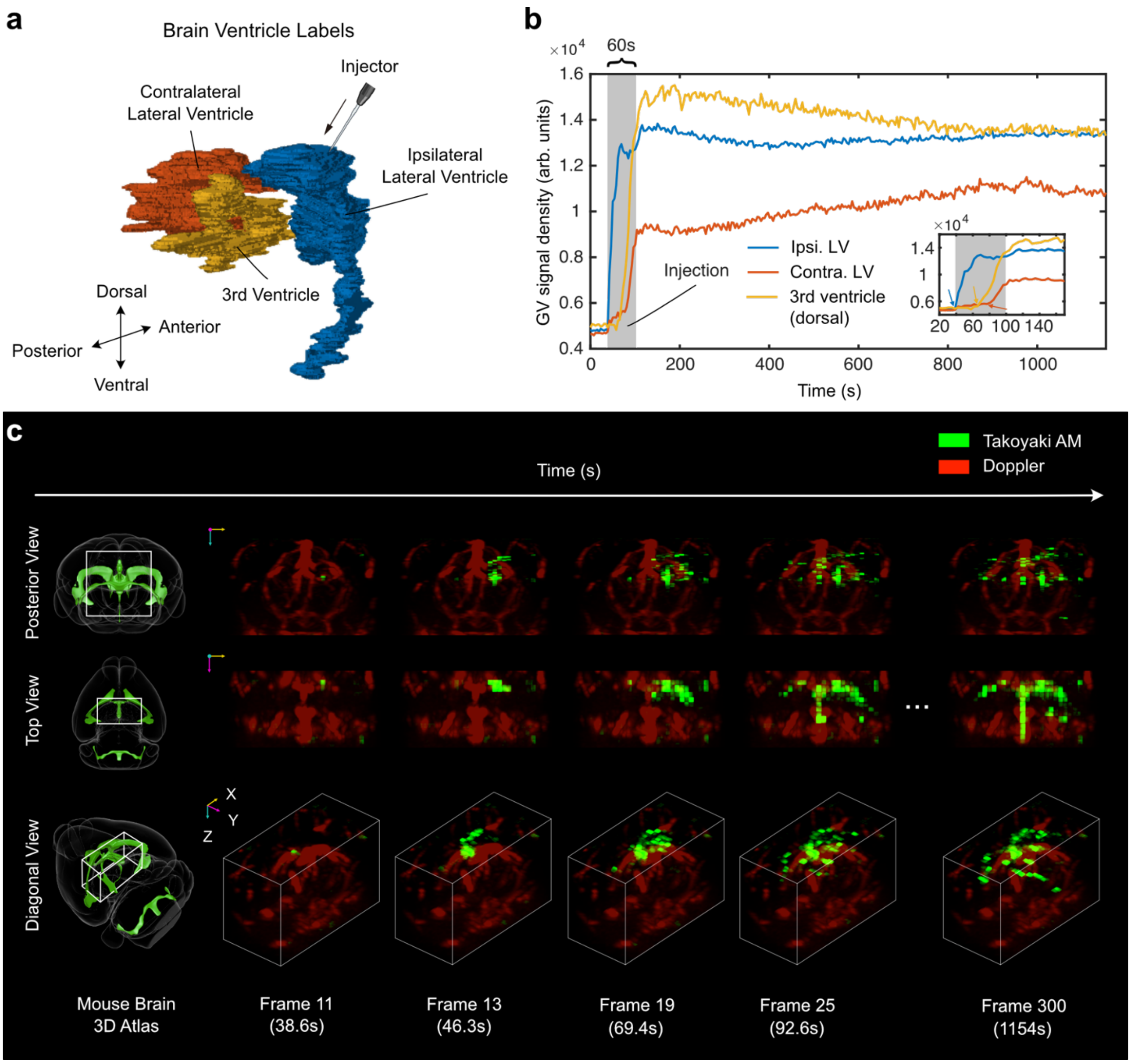


**Supplementary Figure 8: Additional example of monitoring GV propagation in brain ventricles. a**. Manually drawn outlines of brain ventricles. Blue, red, and yellow denote the ipsilateral LV to the injection site, contralateral LV, and third ventricle, respectively. Created with BioRender. Shin. G. (2026) https://BioRender.com/9sggxbg. **b**. Temporal evolution of GV signal density. GV injection (gray shaded area) begins between the frame 11 (38.6s) and 12 (42.5s) **c**. Selected frames from stored real-time images, visualized with Napari. Takoyaki AM images (green) are overlaid on power Doppler images (red). Volume images are half-cropped to exclude the skull side. The dimensions of the cropped Takoyaki AM image are (X, Y, Z) = 7.5 x 4.2 x 6.0 mm, with a depth range from 3.3 mm to 9.3 mm. The sampling interval was approximately 3.8 sec. The 3D Allen Mouse Brain Atlas in the leftmost column illustrates brain orientations. White bounding boxes show the imaging location. Colored arrows have a length of 1 mm along their corresponding directions. Source data are provided as a Source Data file.

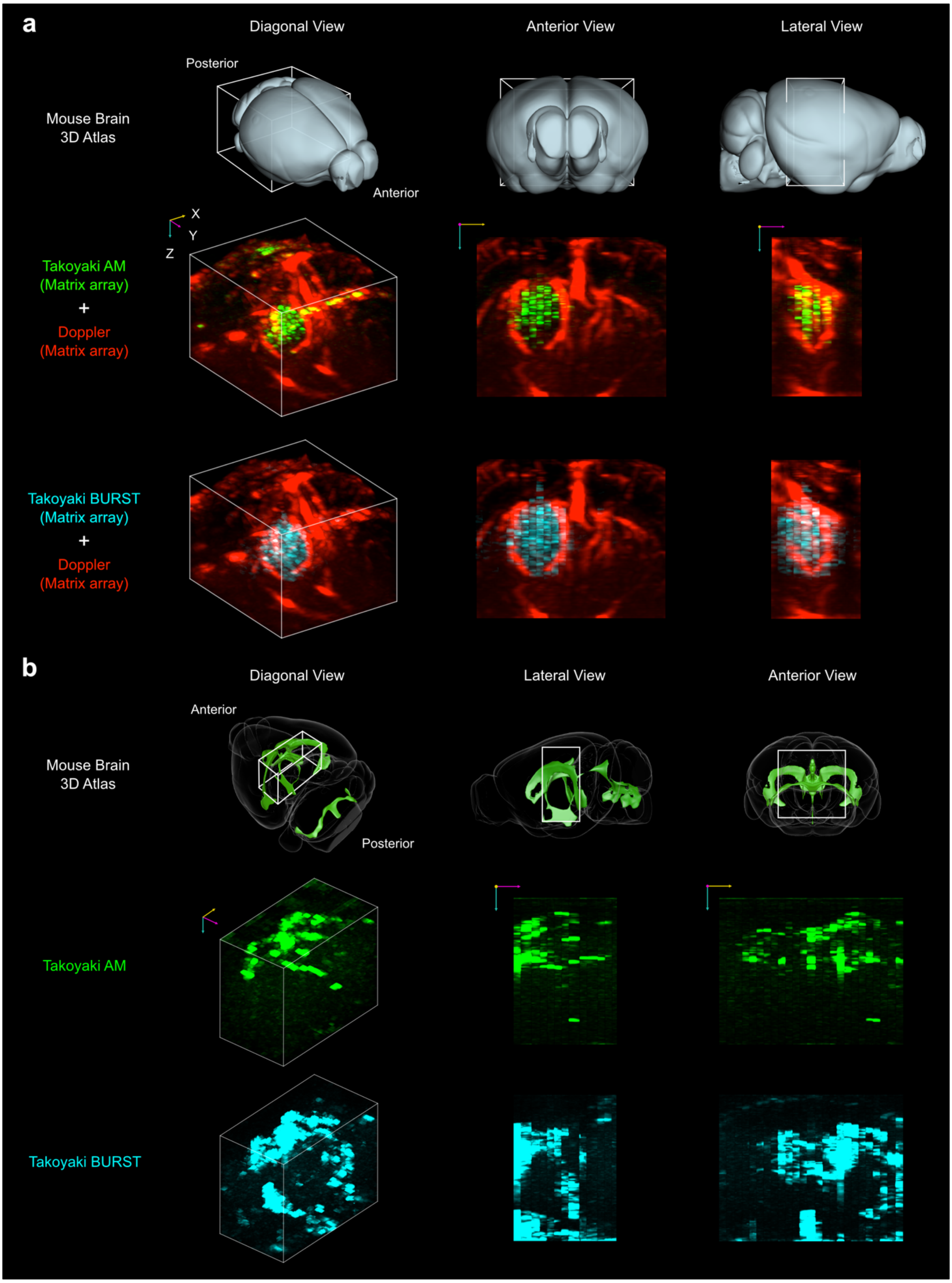


**Supplementary Figure 9: Additional examples of Takoyaki AM (green) and BURST (cyan) images of (a) genetically labeled tumors and (b) brain ventricles. a**. The dimensions of the volume images (left column) is (X, Y, Z) = 7.5 x 8.4 x 5.9 mm, with a depth range from 3.4 mm to 9.3 mm. Power Doppler images (red) are overlaid. The skull areas are excluded in the anterior and lateral views. **b**. Takoyaki AM images from the last frame of real-time recording session (top row) and Takoyaki BURST images (bottom row) acquired after real-time recording. The dimensions of the volume images (left column) are (X, Y, Z) = 7.5 x 4.2 x 6 mm, with a depth range from 3.3 mm to 9.3 mm. The skull areas are excluded in all views. The white box indicates the approximate imaging regions. Colored arrows represent the axes, with lengths of 1 mm.

## Supplementary Note 1: Guidance on Choosing Subaperture Sizes and Focal Length

Although the final decision on parameter selection may require numerical simulation and further optimization, we can provide a starting point based on several main factors. For simplicity, we assume a matrix array with 32 x 32 elements and no inter-bank gaps.

The subaperture size ($N$ x $M$ elements for a single focused beam; $M \leq 8$ along the Y axis) is tied to the FOV and the maximum focal pressure. Assuming that the pressure is sufficient with an $N$ x $M$ subaperture and that the lateral width of the cuboid reconstruction region for a focus is the element pitch $p$, the corresponding FOV (X x Y) is the full array FOV minus the boundary region inaccessible to the focused beam: ($32p$ – $(N-1)p$) x ($32p$ – $(M-1)p$). Moreover, if the delay function has a period of N along the X axis and 8 for the Y axis, the number of different transmit apertures required to cover the FOV is $8N$. For example, when $N = M = 8$ and $p = 0.3$ mm, the FOV is 7.5 x 7.5 mm and the number of different transmit apertures is 64.

The depth of interest can also be a major factor to consider, and predicting the focal zone location helps in choosing the focal length $F$ and the subaperture size. For an ($N$ x $M$)-element rectangular aperture, the transition distances to the focal zone are

$$z_{t_{1,2}} = \frac{z_t F}{z_t \pm F} \tag{1}$$

where $z_{t_1}$ and $z_{t_2}$ are the near and far transition distances, respectively, and $z_t \approx p^2 NM/\pi\lambda$ is the transition distance for the non-focusing case[6]. For instance, when $\lambda = 98.6$ μm, $F = 7$ mm, and other parameters are as given in the previous example, $z_{t_1}$= 5.09 mm and $z_{t_2}$= 11.2 mm. Another useful information is the beam width $w$ of the focused beam,

$$w \sim \frac{F}{a}\lambda \tag{2}$$

where $a$ is the aperture size ($pN$ or $pM$). Under the above conditions, $w$ is approximately 0.29 mm.

## Supplementary Note 2: Estimation of Flow Speed in the Ventricular System and Definition of "Sufficiently Good" Snapshot

Although the precise velocity of injected GVs within the ventricular system is difficult to determine, we can estimate its range from the injection rate (75 nL $s^{-1}$) and ventricular dimensions. Within our FOV, one of the fastest flow regions is likely the interventricular foramen, a narrow passage connecting the lateral and third ventricles. Based on the Allen Mouse Brain Atlas, we estimate its average diameter at ~250 μm. Dividing volumetric flux by the cross-section area, this gives an approximate flow speed of ~1.53 mm $s^{-1}$. Therefore, in most ventricular regions, the flow speed is expected to be below ~1.5 mm $s^{-1}$.

For the definition of a "sufficiently good" snapshot, one can consider a criterion based on the assumption of quasi-static acquisition by requiring that motion during one frame be smaller than a resolution element:

$$|v| * T_{acq} < R \tag{3}$$

where $v$ is velocity, $T_{acq}$ the acquisition time, and $R$ the resolution. Assuming $R$ = 300 μm (**Table 1**) and a PRF of 4 kHz, $T_{acq}$ is 192 msec for Takoyaki imaging. This corresponds to a velocity limit of 1.57 mm $s^{-1}$, which is sufficient to consider the majority of induced ventricular flow (≤ ~1.5 mm $s^{-1}$) as quasi-static during each frame acquisition.

## REFERENCES


1. McCall, J. R., Chavignon, A., Couture, O., Dayton, P. A. & Pinton, G. F. Element Position Calibration for Matrix Array Transducers with Multiple Disjoint Piezoelectric Panels. *Ultrason. Imaging* **46**, 139–150 (2024).
2. Chavignon, A. *et al.* 3D Transcranial Ultrasound Localization Microscopy in the Rat Brain With a Multiplexed Matrix Probe. *IEEE Trans. Biomed. Eng.* **69**, 2132–2142 (2022).
3. Xing, P. *et al.* Towards Transcranial 3D Ultrasound Localization Microscopy of the Nonhuman Primate Brain. Preprint at https://doi.org/10.48550/arXiv.2404.03547 (2024).
4. Hurt, R. C. *et al.* Genomically mined acoustic reporter genes for real-time in vivo monitoring of tumors and tumor-homing bacteria. *Nat. Biotechnol.* **41**, 919–931 (2023).
5. Lakshmanan, A. *et al.* Acoustic biosensors for ultrasound imaging of enzyme activity. *Nat. Chem. Biol.* **16**, 988–996 (2020).
6. Szabo, T. L. *Diagnostic Ultrasound Imaging: Inside Out*. (Academic Press, 2013).
