# Supplementary material for: Real-time volumetric imaging of cells and molecules in deep tissues with Takoyaki ultrasound
Source: Nat Commun. 2026 May 21;17:7281. doi: 10.1038/s41467-026-72961-0 (PMC13402839; doi:10.1038/s41467-026-72961-0)
Supplement: Supplementary file 3 — Description of additional supplementary files [file 41467_2026_72961_MOESM3_ESM.pdf]

Supplementary Movie 1: 3D images of GV phantoms acquired with Takoyaki AM, Sheet-pAM, and 3D xAM, corresponding to **Fig. 2c-e**. Magenta, yellow and cyan arrows represent the X, Y, and Z axes, respectively.

Supplementary Movie 2: 3D images of genetically labeled tumors in the mouse brain, corresponding to **Fig. 3d-e**. Takoyaki AM and st-xAM images are overlaid with a power Doppler image. Magenta, yellow and cyan arrows represent the X, Y, and Z axes, respectively.

Supplementary Movie 3: Real-time recording of nanoparticle transport within mouse brain ventricles, corresponding to **Fig. 4d**. The frame rate is accelerated 20 times. Yellow, magenta and cyan arrows represent the X, Y, and Z axes, respectively. The length of each arrow corresponds to 1 mm.

Supplementary Movie 4: 3D images of genetically labeled tumors and brain ventricles acquired with Takoyaki AM and BURST, corresponding to **Fig. 5b-c**. Magenta, yellow and cyan arrows represent the X, Y, and Z axes, respectively.
