# Supplementary material for: Real-time volumetric imaging of cells and molecules in deep tissues with Takoyaki ultrasound
Source: Nat Commun. 2026 May 21;17:7281. doi: 10.1038/s41467-026-72961-0 (PMC13402839; doi:10.1038/s41467-026-72961-0)
Supplement: Supplementary file 8 — Transparent Peer Review file [file 41467_2026_72961_MOESM8_ESM.pdf]

nature portfolio

Peer Review File

Real-time volumetric imaging of cells and molecules in deep tissues with Takoyaki ultrasound

Corresponding Author: Professor Mikhail Shapiro

This file contains all reviewer reports in order by version, followed by all author rebuttals in order by version.

This manuscript has been previously reviewed at another journal that is not operating a transparent peer review scheme. This document only contains reviewer comments and rebuttal letters for versions considered at Nature Communications.

Version 0:

Reviewer comments:

Reviewer #2

(Remarks to the Author)
We thank the authors for answering a large part of our comments. The authors have made an excellent effort in addressing them, and the paper has undeniably improved.

However, and while the paper is enjoyable to read and visually attractive, the rebuttal did not lift our major concerns (a number of which is shared by several reviewers):

Novelty:
IN our view, the rebuttal confirms that the technique is a specific implementation of multibeam/multiline imaging to 3D, and implementations in 3D have been reported before. The proposed implementation specifically tackles multiplexed arrays, but while this solves an interesting technicality, it does not, in my view, bear broad interest for the wide readership of Nature Communications. The GVs, genetic encoding, nonlinear sequence, etc… have otherwise already been published by the authors. The sheet microscopy technique has been published by others.

Impact/Applicability:
In the rebuttal, the authors explain that this sequence is necessary for GVs because they require high pressures. Therefore, the scientific/technical interest is limited to the use of 2D multiplexed matrix arrays in combination with GVs, and even with nonlinear imaging with GVs. While this is undoubtedly an interesting agent and combination, it is also very niche, and not broadly used. To the concern about the added value of the technique in terms of imaging results, the authors point out that it allows performing functional imaging with GVs (at high pressure), but it remains unclear what this strategy for functional imaging brings with respect to the various ultrasound-based functional imaging techniques that have been (and are being) developed (without GVS). It thus remains unclear what the technique brings from a biology/application perspective.

Position:
The authors interchangeably motivate work on small animals, and clinical use. Yet, clearly, 7 mm is not deep in terms of clinical imaging. The argumentation and explanations restrict the applicability to pre-clinical imaging with small animals (depth, bioengineered cells, frequency, numerical aperture, etc…). How this study should be positioned in terms of clinical imaging has not been further explained. While pre-clinical, small animals imaging is interesting and valuable, it entails different requirements, context, and motivations and, therefore, a different position of the paper to provide an impactful message.

I am therefore sorry to report that I cannot recommend publication in Nature Communications. In my opinion, this paper is better suited for a more specialized technical review.

(Remarks on code availability)

Reviewer #3

(Remarks to the Author)
This manuscript presents an accelerated volumetric imaging approach compatible with acoustic contrast agents requiring high transmit pressure (e.g., gas vesicles) and demonstrates this method in vivo. The authors have provided improved context for the proposed method with regards to the state of the art, especially in justifying why existing wide-field imaging methods are insufficient for the gas vesicle contrast agents used.

I think it would further clarify to divide the Takoyaki focusing configuration into two aspects: 1) multiline transmission from non-overlapping subapertures, compatible with a linear-style probe with large directional elements, and 2) subaperture design to respect the 1024-MUX constraints. Both are novel in the literature, to my knowledge, and perhaps have different levels of interest to different audiences. For example, the MLT approach may be valuable even on a fully populated 1024 channel system.

My main concern is that the manuscript does not consider direct comparison to a single-beam focused beam sequence, only comparing to other rapid acquisition techniques in xAM and sheet-pAM. The proposed sequence, limited by the 4:1 receive aperture multiplexing requiring 4 transmissions per focal configuration, would most directly be compared to a single line focused beam sequence that uses the same focal configuration (i.e., 8x8 subaperture). The proposed 4x4 MLT sequence accelerates the acquisition 16x compared to single line, but given the element directivity a single line would not require all 1024 elements on receive (just the elements nearest the focal spot) so potentially this is actually a 4x speedup over single focused beam imaging if the multiple sparse apertures are not required. Furthermore, in the demonstrated "real-time" application, where reconstruction took 2.8 sec/frame and can be accomplished asynchronously from acquisition on the Verasonics, it seems that line-by-line acquisition could provide similar results. That is, acquisition would take four times longer but displayed volume rate would not change. It seems like this would be sufficient for contrast imaging, but perhaps this would be too slow to represent a "snapshot" in the demonstrated dynamic imaging? Please more clearly justify the need for the more rapid acquisition.

Another possibility for nonlinear imaging (not BURST) is to perform the xAM or Sheet-pAM in both directions to overcome their anisotropic results. This would still require fewer transmissions than the Takoyaki method but you would need to devise a way to combine the results.

Specific comments:

Abstract - "opaque" should perhaps be "optically opaque" to prevent any ambiguity between optics/ultrasound in the discussion
Abstract - "existing ultrasound methods to image acoustic contrast agents predominantly focus on 2D planar imaging" - this is not true of microbubbles, which have been fairly extensively studied with matrix arrays at this point. For example, https://doi.org/10.1109/TMI.2018.2890358. Maybe this is true of gas vesicles and other contrast agents requiring higher pressures
L10 - "planar transducer arrays" may be clearer as "matrix transducer arrays", as planar most commonly refers to planar (2-D) imaging
L30 - the presence of gaps is not inherent to multiplexed arrays, just this particular system (constructed using 4 panels that also correspond to the multiplexer wiring)
L33 "insufficient pressure ranges" - you later provide citations for this (L95) - could you move some of this information up front to clarify the system design requirements?
L55 - "vertical" should maybe be "axial"
L55 - Is there a reason the sequence was designed to shift in one direction, somewhat truncating the field of view on one side, rather than using positive and negative shifts (i.e., +/- 4 rather than +8 shifts)?
Fig 2 - what are the "number of detected peaks" in the caption here? For example the X and Y patterns were 1 mm between peaks so <10 should be observed. Is this across multiple tests?
Table 1 - It would help to add the number of transmissions from the text to this table as well
L158 - Over what time scale would visualizations be valuable biologically (volume rate and total duration)? This could better justify the imaging choices made.
L264 - "compounded receive signals" - should this be "combined" or "accumulated", since the four sets are disjoint? Compounded has connotations of incoherent or coherent summation. Same on L274, L290, L297
Supplement - guidance on choosing subaperture sizes. Could you explain the (33-N)x(36-M) stated field of view? It is not clear why these are asymmetric (if due to the array gaps, those are interior and should not affect FOV), which corresponds to which direction, and why 36 (given 32 elements in each direction). Additionally, the beam width calculated should be 0.29 mm not um.

Verasonics markets a "dynamic HvMUX" model that allows for the creation of non-contiguous apertures. Is that relevant for the proposed method, which appears to use fixed sets of combined transmit elements (one per bank) or would it relax the constraints at all?

(Remarks on code availability)
The code combined with the large data repository hosted on CaltechDATA seem sufficient to reproduce both the method and analysis performed in the paper. The acquisition scripts appear sufficient to run on the Verasonics given appropriate hardware (I cannot test this directly). The analysis code is a mix of MATLAB and Python scripts, presumably to make use of the Napari viewer library in Python.

Version 1:

Reviewer comments:

Reviewer #2

(Remarks to the Author)
At the request of the editor, I accept the argument with respect to novelty. I also thank the authors that have made a few good points in that regard in the last reply. I also wholeheartedly apologize for the lengthy waiting for this report, which I wish I could have done sooner. In this last review report on the paper, I will then go through the answer to the prior comments and further gladly leave the process to the editor and the authors. I thus allow myself to congratulate the authors on the study.

The only remaining (and recurring) point with the previous rebuttal thus is the position should be better addressed. As written before, I find pre-clinical imaging is perfectly acceptable. On the other hand, there is a sizable gap in term of clinical implementation (and of course I agree it could be done provided sufficient work is done in that direction). I allow myself to cite the argument the authors use to address technical concerns: “we agree that further investigation with different hardware and frequencies should be considered in future studies. Nonetheless, 15 MHz ultrasound provides higher resolution than lower-frequency alternatives, which is beneficial for molecular and cellular imaging in small animal models – the current testing ground for most molecular ultrasound approaches”. The authors cannot have it both ways. Amongst others, penetration depth is low, frequency is high. It is not trivial whether a low imaging frequency associated with a lower intrinsic resolution would give the same results. As it stands, this work is suited for pre-clinical work, and I fail to see the point of misleadingly introducing it otherwise when (1) this is perfectly acceptable, and (2) there is a wide gap to at least acknowledge. This would only entail a rather moderate change in the introduction.

In the following, I only reproduce concerns for which the answer does not seem satisfactory. Obviously, since the editorial decision was not in line with my initial advice, I did not give detailed comments. However, I will not start this process at this stage and will trust the other reviewers have covered the essentials. I therefore stick here, to the rebuttal itself. I was also asked to look at the answer from reviewer 1, which I start with:

It is not clear in the paper if a such complex sequence is required or if adjusting few parameters (voltage, transducer frequency, hardware) could alleviate the design of the sequence. In particular, a very simple sequence with a linear probe provides a better sensitivity/resolution and may be enough for molecular imaging of GVs in tumors.

Thank you for your comments. While many details have been addressed throughout our responses to specific remarks below, we summarize the key points here:
• The Takoyaki sequence can generate ultrasound at higher pressure than other unfocused-beam-based sequences when operating at the same voltage. Given the voltage limitations for probe damage, the ability to achieve a wider pressure range within a limited voltage is beneficial for non-linear imaging schemes that require higher pressures, such as BURST, which cannot be achieved with a simple unfocused-beambased sequence.
• We agree that for imaging static tumors, mechanically sweeping a linear probe, which provides better sensitivity and resolution than a matrix array probe at this time, may be enough. However, motion artifacts from breathing or heartbeats (which vary with tumor location) could pose challenges, and a sliding linear array probe has clear limitations in such cases. Additionally, for 3D dynamic imaging, mechanically sweeping introduces large temporal “blurring” within each volume, whereas a matrix array probe does not.

I agree with what the authors write. The comment, however, is not limited to unfocused sequences, but ask about “very simple sequence with a linear probe”. Likewise, there is not (yet) an explicit remark about a sweeping procedure. In short, why is the complex sequence in 3D required for “molecular imaging of GVs in tumors”? Is the answer of the authors that 3D imaging would be unaffected by motion, breathing or heartbeats?

The article mainly presents a very specific ultrasound sequence adapted to one particular multiplexed system. The focalization is used to increase the pressure to activate GV. What are the pressure conditions to activate GV? Is it possible to activate them by increasing the energy of pulses (voltage, length of cycle)? If yes, could it be suitable to use plane wave imaging, or fatbeams to drastically reduce the number of apertures by frames?

These pressure levels cannot be achieved with plane wave imaging, even if voltage and number of cycles are increased within safety limits.

Since the authors insist on clinical application, this seems directly contradicted by Figure S4, provided that the frequency is reduced to the usual imaging range (in any case necessary for resolving the limited penetration) and that the voltage is then increased since lower frequency probes also tolerate higher voltages. The same holds for the following comment, which I therefore do not reproduce here.

The selected hardware (multiplexed 1-4) introduces a complexity in designing the aperture schemes. Using fully addressed

systems would simplify the acquisition scheme (for example 4xVantage by Verasonics, 1024TX-256RX by us4US, SARUSsystem). Also, considering the temporal resolution expected for molecular imaging, could linear probe with scanning system would be suitable (with or without tomographic reconstruction)?

Thank you for this valuable point. It is true that fully addressed 1024-channel systems offer more degrees of freedom in sequence design, which may simplify sequence complexity. However, our study specifically focuses on a multiplexing system, which is much more affordable and portable than a fully addressed system (which we, for example, cannot afford!L). We have also updated the manuscript to state more clearly that our primary focus is the use of a multiplexed matrix probe, to create a method usable by more labs (line 37).
With regard to temporal resolution, mechanically sweeping linear probes introduces substantial temporal heterogeneity into the volume images along the sweeping axis, making this approach not ideal for accurately capturing dynamic phenomena. (Please also see our response to the specific remark on L147)

I consider this a rather minor thing given the scope of the paper, but this answer lacks scientific grounds. What timescale is required for the target application? There is a number of possibilities for "sweeping" 2D probes. Why is a wobbler, for example, not sufficient? The answer lies thus in the timescale that must be reached, which is not discussed.

The experimental set-up for GV injection in ventricles is really interesting. Could it be possible to perform this imaging with conventional ultrasound contrast agents (microbubbles)? As GVs are flowing in the cerebrospinal fluid, how could you ensure that they are homogeneously mixed in the fluid?

Thank you for these questions. To our knowledge, there is no existing literature on injecting microbubbles into mouse brain ventricles. However, according to the Allen Mouse Brain Atlas, the estimated minimum diameter of the Foramen of Monro in mice is roughly 100 µm, making it theoretically possible to use small-diameter microbubbles. Since our main objective here was to determine whether the Takoyaki sequence enables dynamic imaging, the choice of GVs or microbubbles is not critical.

Respectfully, it clearly is. If this can be done with bubbles then the sequence, which is made necessary by the use of GVs, becomes irrelevant. However, this is not crucial, and I am happy to accept the argument (which the authors probably meant to provide) that the increased stability of the GVs is added-value enough. (The same applies to the later comment "L141, why not use a conventional ultrasound contrast agent (microbubbles)? Is nonlinear imaging easier with it?")

L121, reference 45 is not detailed, provide more info or remove it.

This reference is related to the GV-expressing brain tumor model, and the paper will be released soon.

This reference, now number 50, still does not exist. One cannot refer to non-existent papers. Again, please remove it.

L123, the skull was removed, could it be applicable to transcranial imaging?

Thank you for the question. Although in this paper we removed the skull to maximize GV detection inside the brain, we believe it is also applicable to transcranial imaging by increasing the pressure to compensate for the skull's attenuation. We have revised the wording in the original manuscript to avoid implying that skull removal is necessary for imaging (lines 135-136).

Transcranial imaging is not trivial matter, especially since the authors insist on the clinical applicability. Attenuation, distortions, and reverberations require consideration, and simply cranking up the pressure is unlikely to cut it, especially at 15 MHz.

The state of the art in the introduction is largely lacking. There again, much has been done in 3D brain imaging, and functional brain imaging. Result results in super-localization show, a priori, striking results with a better resolution and/or sensitivity. This raises the question of the true novelty/impact of the work: what do the authors do that other techniques cannot, or at least, what do they do better? What is the true value of these results?

We appreciate this feedback. In the revised introduction, we have more clearly mentioned that many state-of-the-art sequences (including those developed for 3D functional brain imaging and super-localization, which are cited as refs. 22-29) mainly used plane waves (lines 32-34). In contrast, our sequence can reach much higher pressure, enabling nonlinear imaging of contrast agents that require high pressure levels.

I thank the authors for this answer. The question, however, was aimed at supporting the (pre-)clinical value of the work. Thus, not whether other techniques can be used with the authors' agents, but rather what is better in the authors technique (system+pulses+agents) as compared to the existing ones (system +pulses+agents).

The authors claim “deep” and “real-time”. This also seems vastly overstated: 7 mm does not qualify as deep, and the approach appears to be as real-time as other techniques, considering the authors need to use SVD filtering. (Anecdotally, the lengthy description of rather standard SVD filtering could be shortened and make room for more valuable information). In that respect, I am also not convinced of the value of creating a new (and rather uninformative) name for the translation from 2D to 3D of an established technique, although I fully understand the marketing value. If I have overlooked novelty aspects with respect to the pulsing schemes, please make them explicit. The title (and some claims in the paper) do not seem to reflect the work appropriately.

Thank you for the comment. In in vivo molecular and cellular imaging, which has been dominantly performed with optical methods, we think 7-10 mm is deep, and this is important to state for general readers (who come from outside the ultrasound field). We have clarified in the main text that “deep” refers specifically to molecular and cellular imaging (lines 14-17). SVD filtering was applied to BURST processing but not to real-time nonlinear imaging. We believe the name “Takoyaki” effectively provides a good visual representation of how we adapted 3D MLT to a multiplexing system, and the community has responded positively to the name.

With respect to the imaging depth, I believe I understand better the authors. The point, however (and this starts to feel quite redundant), is that either the paper is positioned as a potential clinical or preclinical tool for humans, and this is superficial, or it is positioned as a tool for small animal imaging, and the depth is fine. I have no issue with either, but the authors must make a choice.

(Remarks on code availability)

Reviewer #3

(Remarks to the Author)
The revisions have made clear that the main contribution is the development of a volumetric ultrasound imaging approach ("takoyaki" pulse sequencing) that uses multiline transmission on a multiplexed matrix array to achieve sufficient pressure for nonlinear gas vesicle (and possibly other related contrast agents) imaging. This enables "real-time" (~1 fps depending on settings) visualization of nanoparticle transport in mouse brain ventricles and tumor visualization. The contrast agent (GVs) and nonlinear imaging schemes have been demonstrated previously in planar imaging. The technical advance is showing how to perform multiline transmit imaging on a multiplexed matrix array (compared to the cited fully-sampled matrix array approaches published by others previously) for nonlinear imaging. While the advance to volumetric imaging is somewhat incremental, I see this paper being impactful as the work that shows the most clinically relevant (3D) imaging technique if nonlinear contrast agents (esp. gas vesicles) become more common.

The preclinical work should be clear in the abstract - add "mouse" to "dynamics of fluid motion in brain ventricles".

The authors have otherwise addressed my concerns with the manuscript.

(Remarks on code availability)
Code was reviewed previously and satisfactory

Open Access This Peer Review File is licensed under a Creative Commons Attribution 4.0 International License, which permits use, sharing, adaptation, distribution and reproduction in any medium or format, as long as you give appropriate credit to the original author(s) and the source, provide a link to the Creative Commons license, and indicate if changes were made.
In cases where reviewers are anonymous, credit should be given to 'Anonymous Referee' and the source.
The images or other third party material in this Peer Review File are included in the article's Creative Commons license, unless indicated otherwise in a credit line to the material. If material is not included in the article's Creative Commons license and your intended use is not permitted by statutory regulation or exceeds the permitted use, you will need to obtain permission directly from the copyright holder.
To view a copy of this license, visit https://creativecommons.org/licenses/by/4.0/

We again appreciate the reviewers' constructive comments, which helped us improve the manuscript. Our responses to reviewer comments are provided below in blue font. Corresponding changes in the manuscript and supplementary material are also highlighted in blue, and a new Supplementary Note 2 has been added.

Reviewer #2 (Remarks to the Author):

We thank the authors for answering a large part of our comments. The authors have made an excellent effort in addressing them, and the paper has undeniably improved.

However, and while the paper is enjoyable to read and visually attractive, the rebuttal did not lift our major concerns (a number of which is shared by several reviewers):

Novelty:
IN our view, the rebuttal confirms that the technique is a specific implementation of multibeam/multiline imaging to 3D, and implementations in 3D have been reported before. The proposed implementation specifically tackles multiplexed arrays, but while this solves an interesting technicality, it does not, in my view, bear broad interest for the wide readership of Nature Communications. The GVs, genetic encoding, nonlinear sequence, etc... have otherwise already been published by the authors. The sheet microscopy technique has been published by others.

Thank you for the valuable comment. We believe that, as one of the first 3D visualizations of a rapidly emerging ultrasound contrast agent – GVs – our study will be interesting to a wide range of readers, including cellular/molecular biology and ultrasound imaging. While the nonlinear sound sheet imaging (NSSI; ref. 40 in the manuscript)[1] also demonstrated 3D imaging of GVs, the paper was essentially contemporaneous with ours (3.5 months earlier; NSSI: 2024-07-31, Takoyaki: 2024-11-14 on Biorxiv) and used a different probe configuration (2D RCA array); matrix-array-based volumetric imaging of GVs had not been previously done. In addition, our work uniquely demonstrates GV-based imaging of dynamic processes (perfusion in brain ventricles) as well as volumetric BURST imaging, which have not been shown in prior studies. BURST imaging is especially valuable as it has formed the basis of recent advances in imaging cell-based diagnostic and therapeutic agents[2,3].

Impact/Applicability:
In the rebuttal, the authors explain that this sequence is necessary for GVs because they require high pressures. Therefore, the scientific/technical interest is limited to the use of 2D multiplexed matrix arrays in combination with GVs, and even with nonlinear imaging with GVs. While this is undoubtedly an interesting agent and combination, it is also very niche, and not broadly used. To the concern about the added value of the technique in terms of imaging results, the authors point out that it allows performing functional imaging with GVs (at high pressure), but it remains unclear what this strategy for functional imaging brings with respect to the various ultrasound-based functional imaging techniques that have been (and are being) developed (without GVS). It thus remains unclear what the technique brings from a biology/application perspective.

Thank you for your valuable comment. We agree that, compared to microbubbles, GVs are not yet as broadly used; however, they represent a major emerging field with a growing number of

publications in recent years, along with the increasing use of matrix array transducer (Figure 1 below). We expect this trend will continue. We believe the value of the technique lies in enabling volumetric imaging of contrast agents that require high acoustic pressures while maintaining high temporal coherence, using a multiplexed matrix array system (more accessible and affordable than a full 1024-channel system). This capability distinguishes our method from most of the other various functional 3D imaging techniques that have been developed (without GVs). From a biological/application perspective, the scope of new functional imaging enabled by this technique mainly depends on the unique capabilities of these contrast agents – not only GVs, but potentially also nanodroplet[4] and nanobubbles[5]. In this work, we demonstrated several important GV-based applications such as tumor imaging and ventricular perfusion imaging. Looking ahead, the applications of this volumetric imaging technique can always be expandable in parallel with GV development – including but not limited to intra-cellular calcium imaging[6], probiotic biosensors of gastrointestinal inflammation[2], and T-cell activation monitoring[3].

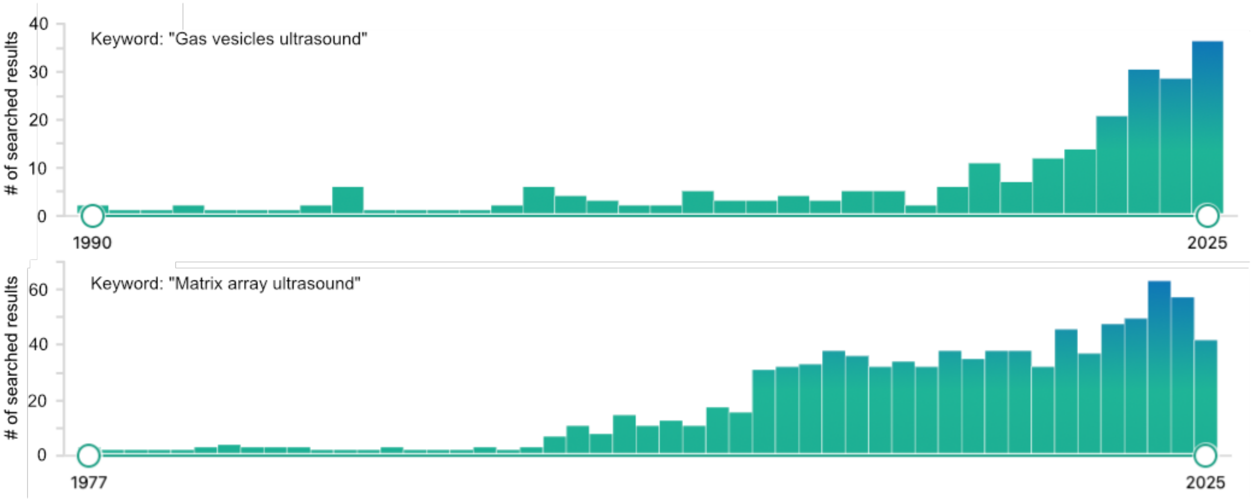

Figure 1. The annual number of publications related to "gas vesicles ultrasound" (Top) and "matrix array ultrasound" (Bottom) (PubMed)

Position:
The authors interchangeably motivate work on small animals, and clinical use. Yet, clearly, 7 mm is not deep in terms of clinical imaging. The argumentation and explanations restrict the applicability to pre-clinical imaging with small animals (depth, bioengineered cells, frequency, numerical aperture, etc...). How this study should be positioned in terms of clinical imaging has not been further explained. While pre-clinical, small animals imaging is interesting and valuable, it entails different requirements, context, and motivations and, therefore, a different position of the paper to provide an impactful message.

Thank you for the comment. Similar to many imaging techniques published in Nature Communications[7–9], our work primarily focuses on pre-clinical small-animal studies, which are essential for advancing fundamental biological and biomedical research. Nonetheless, this focus does not preclude clinical translation. By using a matrix array with a lower center frequency (ideally combined with a larger aperture) and adjusting the focal length, Takoyaki imaging could be adapted for deeper tissue visualization in clinical contexts. Moreover, GV imaging has been demonstrated at lower frequency[10–12], and gene delivery techniques enabling native tissue to express GVs are being developed[13]. Taken together, these efforts make the clinical application of our approach highly

promising. We have revised the manuscript to clarify the potential of Takoyaki imaging for clinical applications in the main text (L226-228).

I am therefore sorry to report that I cannot recommend publication in Nature Communications. In my opinion, this paper is better suited for a more specialized technical review.

Reviewer #3 (Remarks to the Author):

This manuscript presents an accelerated volumetric imaging approach compatible with acoustic contrast agents requiring high transmit pressure (e.g., gas vesicles) and demonstrates this method in vivo. The authors have provided improved context for the proposed method with regards to the state of the art, especially in justifying why existing wide-field imaging methods are insufficient for the gas vesicle contrast agents used.

I think it would further clarify to divide the Takoyaki focusing configuration into two aspects: 1) multiline transmission from non-overlapping subapertures, compatible with a linear-style probe with large directional elements, and 2) subaperture design to respect the 1024-MUX constraints. Both are novel in the literature, to my knowledge, and perhaps have different levels of interest to different audiences. For example, the MLT approach may be valuable even on a fully populated 1024 channel system.

My main concern is that the manuscript does not consider direct comparison to a single-beam focused beam sequence, only comparing to other rapid acquisition techniques in xAM and sheet-pAM. The proposed sequence, limited by the 4:1 receive aperture multiplexing requiring 4 transmissions per focal configuration, would most directly be compared to a single line focused beam sequence that uses the same focal configuration (i.e., 8x8 subaperture). The proposed 4x4 MLT sequence accelerates the acquisition 16x compared to single line, but given the element directivity a single line would not require all 1024 elements on receive (just the elements nearest the focal spot) so potentially this is actually a 4x speedup over single focused beam imaging if the multiple sparse apertures are not required. Furthermore, in the demonstrated "real-time" application, where reconstruction took 2.8 sec/frame and can be accomplished asynchronously from acquisition on the Verasonics, it seems that line-by-line acquisition could provide similar results. That is, acquisition would take four times longer but displayed volume rate would not change. It seems like this would be sufficient for contrast imaging, but perhaps this would be too slow to represent a "snapshot" in the demonstrated dynamic imaging? Please more clearly justify the need for the more rapid acquisition.

We thank the reviewer for this insightful point. If only one receive aperture is used for single-line transmission (SLT), the acquisition time difference with Takoyaki decreases (64 x 4 x 3 vs 625 x 3). However, SLT still suffers from lower intra-frame temporal coherence, compromising the ability to capture an accurate “snapshot” of fast-changing biological processes such as mouse ventricular perfusion.

A practical criterion for snapshot quality is that motion during the acquisition should be less than one resolution element:

$$|v| * T_{acq} < R$$

where v is velocity, T_{acq} is frame acquisition time, and R is resolution. With R = 300 μm and PRF = 4 kHz, T_{acq} is 192 ms for Takoyaki and 469 ms for SLT, corresponding to velocity limits of 1.57 mm/s vs. 0.64 mm/s. Estimated flow speeds in the ventricular system (e.g. ~1.53 mm/s at the foramen of Monro) meet this criterion for Takoyaki but exceed it for SLT. Thus, SLT cannot provide reliable snapshots in our dynamic example. We have added to the main text a comparison to SLT in terms of temporal coherence (L68-70) and a justification for using more rapid acquisition in our dynamic imaging demonstration (L159-160, L170-174, Supplementary Note 2 – Estimation of Flow Speed in the Ventricular System and Definition of "Sufficiently Good" Snapshot)

While reconstruction in our demonstration was limited (~2.8 s/frame), this can be substantially reduced with GPU acceleration, leaving T_{acq} as the dominant factor. Ultimately, Takoyaki enables higher effective frame rates and better captures fast phenomena, including neural activity[6], than SLT.

Higher temporal coherence and frame rate also help mitigate motion artifacts. While our work targeted relatively motionless tissue, other regions (e.g. the abdomen) are more vulnerable to physiological motion such as breathing (~3 Hz); here, Takoyaki's higher temporal coherence helps avoid motion blur, compared to SLT. In addition, BURST imaging benefits directly from Takoyaki's higher frame rate, since the number of post-collapse frames acquired within a motion-free window strongly determines final image quality.

Another possibility for nonlinear imaging (not BURST) is to perform the xAM or Sheet-pAM in both directions to overcome their anisotropic results. This would still require fewer transmissions than the Takoyaki method but you would need to devise a way to combine the results.

Thank you for the valuable comment. As the reviewer stated, if 3D xAM and sheet-pAM are acquired in both directions and combined, the number of transmissions would still be lower than that required for Takoyaki imaging. However, as we mentioned in the main text (L94-97), we did not consider 3D xAM and Sheet-pAM with the alternative orientations (Sheet-pAM imaging YZ planes and 3D xAM imaging XZ planes) or combining with these orthogonal implementations because matrix-array gaps increase pressure-field heterogeneity for sheet-pAM and disrupt the axisymmetry of cross-propagating waves in 3D xAM. The alternate 3D xAM orientation also restricts the X-wave aperture due to the parallel element constraint, which substantially limits imaging depth. Finally, as our goal was to design a sequence that supports both AM and BURST imaging, we did not pursue versions of these sequences that scan the entire volume twice (along both directions) to form a single volume image. We have now clarified in the main text that combining with the orthogonal implementations was not considered for the same reasons mentioned for the alternative orientations (L95-96).

Specific comments:

Abstract - "opaque" should perhaps be "optically opaque" to prevent any ambiguity between optics/ultrasound in the discussion

Thank you for the suggestion. We have changed the expression in the abstract to "optically opaque" to avoid the possible ambiguity.

Abstract - "existing ultrasound methods to image acoustic contrast agents predominantly focus on 2D planar imaging" - this is not true of microbubbles, which have been fairly extensively studied with matrix arrays at this point. For example, https://doi.org/10.1109/TMI.2018.2890358. Maybe this is true of gas vesicles and other contrast agents requiring higher pressures

Thank you for this comment. As we also cited the paper as ref. 25 in the introduction, we agree that the original sentence - "existing ultrasound methods to image acoustic contrast agents predominantly focus on 2D planar imaging" – could be interpreted as overlooking prior work on 3D ultrasound imaging of microbubbles with matrix arrays. We have revised the sentence in the abstract to emphasize that the limited availability of 3D imaging methods specifically pertains to contrast agents that require high acoustic pressures.

L10 - "planar transducer arrays" may be clearer as "matrix transducer arrays", as planar most commonly refers to planar (2-D) imaging

Thank you for the suggestion. We have revised the wording to "2D transducer arrays" to more accurately reflect our intention to include not only matrix arrays but also other types of 2D array probes, such as RCA.

L30 - the presence of gaps is not inherent to multiplexed arrays, just this particular system (constructed using 4 panels that also correspond to the multiplexer wiring)

Thank you for the comment. We agree that the presence of gaps is not an inherent feature of multiplexed matrix arrays, but rather a characteristic of certain commercial implementations where wiring constraints have led to such gaps. We have revised the sentence accordingly to clarify that only some commercialized multiplexed matrix arrays exhibit such gaps (L30-31).

L33 "insufficient pressure ranges" - you later provide citations for this (L95) - could you move some of this information up front to clarify the system design requirements?

Thank you for the valuable suggestion. We have moved some of the pressure information to an earlier point in the main text (L34) to improve clarity on the system design requirements.

L55 - "vertical" should maybe be "axial"

Thank you for pointing it out. We have changed "vertical" to "axial".

L55 - Is there a reason the sequence was designed to shift in one direction, somewhat truncating the field of view on one side, rather than using positive and negative shifts (i.e., +/- 4 rather than +8 shifts)?

Since the focal points are arranged periodically, a negative shift value of -a is equal to a positive shift value of (8-a). For example, shifting aperture 1 (and its focal points) in Fig. 1d by either +1 and -7 along the Y axis yields the same aperture 2 (and its focal points). To simplify the explanation of the sequence, we illustrated the focal points as shifting in only one direction, but the order of apertures

can be permuted according to the user's preferences, as if the focal points were moving with both positive and negative shifts.

Fig 2 - what are the "number of detected peaks" in the caption here? For example the X and Y patterns were 1 mm between peaks so <10 should be observed. Is this across multiple tests?

Yes, the number of detected peaks was counted across 6 phantoms and multiple lines at different locations within the volume images. For example, when measuring lateral resolution along the X axis, peaks were detected from lines at different Y positions across all 6 phantoms (details provided in Methods). We have now clarified in the caption of Fig. 2 that the reported number of detected peaks reflects measurements from 6 phantoms.

Table 1 - It would help to add the number of transmissions from the text to this table as well

Thank you for the suggestion. We have added the number of transmissions to Table 1 and adjusted its title accordingly.

L158 - Over what time scale would visualizations be valuable biologically (volume rate and total duration)? This could better justify the imaging choices made.

Thank you for the comment. The appropriate volume rate and total duration depend on the biological phenomena of interest (e.g. fast/slow processes, long-/short-term). While higher frame rates are generally advantageous, in our dynamic imaging demonstration a rate of 1 frame/3.8 sec was enough to visualize the macroscopic spread of GVs across the brain ventricles (Fig. 4c, d). Regarding frame acquisition time, we have more emphasized the importance of intra-frame temporal coherence (L159-160) and added justification based on estimated flow speed (L170-174, Supplementary Note 2 – Estimation of Flow Speed in the Ventricular System and Definition of "Sufficiently Good" Snapshot), as also discussed in our response to the first general comment above.

L264 - "compounded receive signals" - should this be "combined" or "accumulated", since the four sets are disjoint? Compounded has connotations of incoherent or coherent summation. Same on L274, L290, L297

We appreciate you pointing this out. We have changed "compounded" to "combined" in all of these lines (L272, 282-283, 299, 306).

Supplement - guidance on choosing subaperture sizes. Could you explain the (33-N)x(36-M) stated field of view? It is not clear why these are asymmetric (if due to the array gaps, those are interior and should not affect FOV), which corresponds to which direction, and why 36 (given 32 elements in each direction). Additionally, the beam width calculated should be 0.29 mm not um.

We thank the reviewer for these comments and for identifying the error. We have corrected the unit error from 0.29 µm to 0.29 mm. Regarding the FOV, it was calculated as the full array FOV minus the boundary region inaccessible with a focused beam. Since we used the asymmetric full-array FOV (including gaps) in this calculation, the resulting FOV also appeared asymmetric. To simplify the

explanation, we have added the assumption that the matrix array has no inter-bank gaps. We have also more clearly clarified the calculation process of FOV and indicated which axes correspond to each dimension of the FOV.

Verasonics markets a "dynamic HvMUX" model that allows for the creation of non-contiguous apertures. Is that relevant for the proposed method, which appears to use fixed sets of combined transmit elements (one per bank) or would it relax the constraints at all?

Yes, it is relevant, and our script already uses dynamic HvMUX programming (e.g. “computeMuxAperture()” or “computeUTAMux1024()”), which generates aperture numbers for arbitrary multiplexer apertures. To our knowledge, all Vantage example scripts for multiplexed matrix arrays use HvMUX programming, so we think this is effectively the default when operating multiplexed matrix arrays on the Verasonics system.

Reviewer #3 (Remarks on code availability):

The code combined with the large data repository hosted on CaltechDATA seem sufficient to reproduce both the method and analysis performed in the paper. The acquisition scripts appear sufficient to run on the Verasonics given appropriate hardware (I cannot test this directly). The analysis code is a mix of MATLAB and Python scripts, presumably to make use of the Napari viewer library in Python.

References

1. Heiles, B. *et al.* Nonlinear sound-sheet microscopy: Imaging opaque organs at the capillary and cellular scale. *Science* **388**, eads1325 (2025).
2. Buss, M. T., Zhu, L., Kwon, J. H., Tabor, J. J. & Shapiro, M. G. Probiotic acoustic biosensors for noninvasive imaging of gut inflammation. *Nat. Commun.* **16**, 7931 (2025).
3. Shivaei, S. *et al.* Non-invasive imaging of cell-based therapies using acoustic reporter genes. 2024.11.01.621111 Preprint at https://doi.org/10.1101/2024.11.01.621111 (2024).
4. Dorvashi, M. *et al.* A meta-analysis of the effect of ultrasound activation parameters on phase-change nanodroplets in imaging and therapy. *Front. Acoust.* **2**, (2024).
5. Wegierak, D. *et al.* Nanobubble Contrast Enhanced Ultrasound Imaging: A Review. *WIREs Nanomedicine Nanobiotechnology* **16**, e2007 (2024).
6. Jin, Z. *et al.* Ultrasonic reporters of calcium for deep tissue imaging of cellular signals. 2023.11.09.566364 Preprint at https://doi.org/10.1101/2023.11.09.566364 (2023).
7. Deán-Ben, X. L. *et al.* Deep optoacoustic localization microangiography of ischemic stroke in mice. *Nat. Commun.* **14**, 3584 (2023).
8. Menozzi, L. *et al.* Three-dimensional diffractive acoustic tomography. *Nat. Commun.* **16**, 1149 (2025).
9. Kasatkina, L. A. *et al.* Deep-tissue high-sensitivity multimodal imaging and optogenetic manipulation enabled by biliverdin reductase knockout. *Nat. Commun.* **16**, 6469 (2025).
10. Shapiro, M. G. *et al.* Biogenic gas nanostructures as ultrasonic molecular reporters. *Nat. Nanotechnol.* **9**, 311–316 (2014).

11. Liu, T. *et al.* Characterization and Comparison of Contrast Imaging Properties of Naturally Isolated and Heterologously Expressed Gas Vesicles. *Pharmaceuticals* **17**, 755 (2024).
12. Li, J. *et al.* Surface-engineered bio-manufactured gas vesicles for multimodal imaging of glioma. *J. Nanobiotechnology* **23**, 116 (2025).
13. Shivaei, S. *et al.* Ultrasound imaging of in situ transcriptional activity in opaque tissue. 2025.07.06.663365 Preprint at https://doi.org/10.1101/2025.07.06.663365 (2025).

We appreciate the reviewers' helpful comments, which have enabled us to further improve the manuscript. Our responses to the reviewer comments are provided below in blue font. Corresponding changes in the manuscript are also highlighted in blue.

Reviewer #2 (Remarks to the Author):

At the request of the editor, I accept the argument with respect to novelty. I also thank the authors that have made a few good points in that regard in the last reply. I also wholeheartedly apologize for the lengthy waiting for this report, which I wish I could have done sooner. In this last review report on the paper, I will then go through the answer to the prior comments and further gladly leave the process to the editor and the authors. I thus allow myself to congratulate the authors on the study.

The only remaining (and recurring) point with the previous rebuttal thus is the position should be better addressed. As written before, I find pre-clinical imaging is perfectly acceptable. On the other hand, there is a sizable gap in term of clinical implementation (and of course I agree it could be done provided sufficient work is done in that direction). I allow myself to cite the argument the authors use to address technical concerns: "we agree that further investigation with different hardware and frequencies should be considered in future studies. Nonetheless, 15 MHz ultrasound provides higher resolution than lower-frequency alternatives, which is beneficial for molecular and cellular imaging in small animal models – the current testing ground for most molecular ultrasound approaches". The authors cannot have it both ways. Amongst others, penetration depth is low, frequency is high. It is not trivial whether a low imaging frequency associated with a lower intrinsic resolution would give the same results. As it stands, this work is suited for pre-clinical work, and I fail to see the point of misleadingly introducing it otherwise when (1) this is perfectly acceptable, and (2) there is a wide gap to at least acknowledge. This would only entail a rather moderate change in the introduction.

To clarify that this first publication on Takoyaki imaging is focused on small-animal/pre-clinical studies, we have revised any parts that might imply clinical applications of the current technique, although, as the reviewer also agreed, we believe that, with appropriate adaptations, this imaging mode could be used in larger animals and humans in future studies. We have more clearly emphasized that our target use case in this paper is pre-clinical, molecular, and cellular imaging in small animals throughout the abstract, introduction (L15, 48-49), results (L169 and subheadings), and discussion (L220-221; previous L226-228 removed).

In the following, I only reproduce concerns for which the answer does not seem satisfactory. Obviously, since the editorial decision was not in line with my initial advice, I did not give detailed comments. However, I will not start this process at this stage and will trust the other reviewers have covered the essentials. I therefore stick here, to the rebuttal itself. I was also asked to look at the answer from reviewer 1, which I start with:

It is not clear in the paper if a such complex sequence is required or if adjusting few parameters (voltage, transducer frequency, hardware) could alleviate the design of the sequence. In particular, a very simple sequence with a linear probe provides a better sensitivity/resolution and may be

enough for molecular imaging of GVs in tumors.

Thank you for your comments. While many details have been addressed throughout our responses to specific remarks below, we summarize the key points here:

• The Takoyaki sequence can generate ultrasound at higher pressure than other unfocused-beam-based sequences when operating at the same voltage. Given the voltage limitations for probe damage, the ability to achieve a wider pressure range within a limited voltage is beneficial for non-linear imaging schemes that require higher pressures, such as BURST, which cannot be achieved with a simple unfocused-beam based sequence.

• We agree that for imaging static tumors, mechanically sweeping a linear probe, which provides better sensitivity and resolution than a matrix array probe at this time, may be enough. However, motion artifacts from breathing or heartbeats (which vary with tumor location) could pose challenges, and a sliding linear array probe has clear limitations in such cases. Additionally, for 3D dynamic imaging, mechanically sweeping introduces large temporal "blurring" within each volume, whereas a matrix array probe does not.

I agree with what the authors write. The comment, however, is not limited to unfocused sequences, but ask about "very simple sequence with a linear probe". Likewise, there is not (yet) an explicit remark about a sweeping procedure. In short, why is the complex sequence in 3D required for "molecular imaging of GVs in tumors"? Is the answer of the authors that 3D imaging would be unaffected by motion, breathing or heartbeats?

3D imaging with a 2D array, such as a matrix array, provides significantly higher temporal synchrony within a volume image compared to a mechanically sliding array probe, as we briefly mentioned in the Introduction (L11-12) and the second bullet above ("temporal blurring within each volume"). This high intra-frame temporal synchrony makes the resulting 3D images much less affected by motion. For example, acquiring one Takoyaki AM frame takes 192 msec, whereas a mechanically sliding linear array would require ~5 sec to image a comparable 3D volume, even with automated robotic translation[1] (25 x 0.2 sec movement intervals). Therefore, a sliding linear array probe is inevitably more vulnerable to physiological motion (e.g. breathing at ~1 Hz) when imaging high-motion anatomical locations (e.g. the abdomen), while the Takoyaki sequence is much less susceptible to motion artifacts and can acquire a frame within short motion-free windows. To more explicitly distinguish sliding-linear-array imaging from Takoyaki imaging, we have added more details in the manuscript about how we translated the linear array probe (L147-148, 154-155, 486) and clarified the advantages of the Takoyaki sequence over a sweeping linear array (L154-159).

The article mainly presents a very specific ultrasound sequence adapted to one particular multiplexed system. The focalization is used to increase the pressure to activate GV. What are the pressure conditions to activate GV? Is it possible to activate them by increasing the energy of pulses (voltage, length of cycle)? If yes, could it be suitable to use plane wave imaging, or fatbeams to drastically reduce the number of apertures by frames?

These pressure levels cannot be achieved with plane wave imaging, even if voltage and number of cycles are increased within safety limits.

Since the authors insist on clinical application, this seems directly contradicted by Figure S4, provided that the frequency is reduced to the usual imaging range (in any case necessary for resolving the limited penetration) and that the voltage is then increased since lower frequency probes also tolerate higher voltages. The same holds for the following comment, which I therefore do not reproduce here.

Our response above and Figure S4 assumed the use of a 15-MHz ultrasound matrix array. As we mentioned in our response to the first comment, we have revised the manuscript to clarify that the present study is focused on preclinical applications using 15 MHz (please refer to our response to the first comment for the updated manuscript sections).

The selected hardware (multiplexed 1-4) introduces a complexity in designing the aperture schemes. Using fully addressed systems would simplify the acquisition scheme (for example 4xVantage by Verasonics, 1024TX-256RX by us4US, SARUSsystem). Also, considering the temporal resolution expected for molecular imaging, could linear probe with scanning system would be suitable (with or without tomographic reconstruction)?

Thank you for this valuable point. It is true that fully addressed 1024-channel systems offer more degrees of freedom in sequence design, which may simplify sequence complexity. However, our study specifically focuses on a multiplexing system, which is much more affordable and portable than a fully addressed system (which we, for example, cannot afford!L). We have also updated the manuscript to state more clearly that our primary focus is the use of a multiplexed matrix probe, to create a method usable by more labs (line 37).
With regard to temporal resolution, mechanically sweeping linear probes introduces substantial temporal heterogeneity into the volume images along the sweeping axis, making this approach not ideal for accurately capturing dynamic phenomena. (Please also see our response to the specific remark on L147)

I consider this a rather minor thing given the scope of the paper, but this answer lacks scientific grounds. What timescale is required for the target application? There is a number of possibilities for “sweeping” 2D probes. Why is a wobbler, for example, not sufficient? The answer lies thus in the timescale that must be reached, which is not discussed.

The required timescale depends on the dynamic phenomena being targeted (e.g. the typical frame rate for calcium neural imaging is 1-100 Hz[2]). Considering the adjustable imaging parameters, such as pulse repetition frequency, frame rate, and processing methods, Takoyaki AM imaging is appropriate for dynamics on the order of ~25 Hz, based on its theoretical maximum frame acquisition speed (17.4 msec for a 1-cm imaging depth; L243-245). As we described in our response to the second comment, a sliding linear array would take ~5 sec (~0.2 Hz) to image a similar size of volume, which is too slow to capture the target phenomena for Takoyaki imaging. Using a wobbler, which also mechanically moves a linear array probe, is likewise slower. We have now specified the timescale appropriate for using Takoyaki imaging in the Discussion (L240-241, 245-246).

The experimental set-up for GV injection in ventricles is really interesting. Could it be possible to perform this imaging with conventional ultrasound contrast agents (microbubbles)? As GVs are flowing in the cerebrospinal fluid, how could you ensure that they are homogeneously mixed in the fluid?

Thank you for these questions. To our knowledge, there is no existing literature on injecting microbubbles into mouse brain ventricles. However, according to the Allen Mouse Brain Atlas, the estimated minimum diameter of the Foramen of Monro in mice is roughly 100 μm, making it theoretically possible to use small-diameter microbubbles. Since our main objective here was to determine whether the Takoyaki sequence enables dynamic imaging, the choice of GVs or microbubbles is not critical.

Respectfully, it clearly is. If this can be done with bubbles then the sequence, which is made necessary by the use of GVs, becomes irrelevant. However, this is not crucial, and I am happy to accept the argument (which the authors probably meant to provide) that the increased stability of the GVs is added-value enough. (The same applies to the later comment "L141, why not use a conventional ultrasound contrast agent (microbubbles)? Is nonlinear imaging easier with it?")

As the reviewer mentioned, a key advantage of Takoyaki imaging is that it can leverage the high stability of GVs to provide more persistent contrast over time to visualize temporally evovling phenomena, as we showed with ventricular flow imaging. We have revised the ventricular imaging section to clarify that the use of GVs improves the stability of ultrasound contrast to support extended imaging (L196-198). We have also cited other studies in which GVs were imaged over extended time scales (L198-199).

L121, reference 45 is not detailed, provide more info or remove it.

This reference is related to the GV-expressing brain tumor model, and the paper will be released soon.

This reference, now number 50, still does not exist. One cannot refer to non-existent papers. Again, please remove it.

The preprint of this reference (ref. 50) is now available and we have included the appropriate citation.

L123, the skull was removed, could it be applicable to transcranial imaging?

Thank you for the question. Although in this paper we removed the skull to maximize GV detection inside the brain, we believe it is also applicable to transcranial imaging by increasing the pressure to compensate for the skull's attenuation. We have revised the wording in the original manuscript to avoid implying that skull removal is necessary for imaging (lines 135-136).

Transcranial imaging is not trivial matter, especially since the authors insist on the clinical applicability. Attenuation, distortions, and reverberations require consideration, and simply cranking up the pressure is unlikely to cut it, especially at 15 MHz.

We assumed that transcranial imaging in this question referred to imaging in mice, which has already been demonstrated at 15 MHz in prior studies[3,4]. To emphasize that our work is focused on a pre-clinical scope, as noted in our response to the first comment, we have revised our manuscript that could be interpreted as insisting on clinical applicability (please refer to our response to the first comment for the updated manuscript sections).

The state of the art in the introduction is largely lacking. There again, much has been done in 3D brain imaging, and functional brain imaging. Result results in super-localization show, a priori, striking results with a better resolution and/or sensitivity. This raises the question of the true novelty/impact of the work: what do the authors do that other techniques cannot, or at least, what do they do better? What is the true value of these results?

We appreciate this feedback. In the revised introduction, we have more clearly mentioned that many state-of-the-art sequences (including those developed for 3D functional brain imaging and super-localization, which are cited as refs. 22-29) mainly used plane waves (lines 32-34). In contrast, our sequence can reach much higher pressure, enabling nonlinear imaging of contrast agents that require high pressure levels.

I thank the authors for this answer. The question, however, was aimed at supporting the (pre-)clinical value of the work. Thus, not whether other techniques can be used with the authors' agents, but rather what is better in the authors technique (system+pulses+agents) as compared to the existing ones (system +pulses+agents).

We edited our introduction and discussion to make our rationale and comparisons clearer, emphasizing that our newly-introduced Takoyaki approach enables volumetric imaging of nonlinear contrast agents such as GVs at the higher pressures required for their activation, using a multiplexing system that makes this approach practically accessible to many laboratories. This, in turn, provides access to a wider range of biological functions (conferred by these advanced contrast agents) than is possible with existing ultrasound contrast imaging techniques (or system + pulses + agents, as the reviewer phrased it). These novel functional readouts reveal cellular functions such as gene expression and intracellular signaling that advanced agents like GVs can be engineered to report.

Biomolecular and cellular ultrasound is a transformative research area that is giving unprecedented access to biological phenomena in vivo. We have emphasized the importance of this field, and how Takoyaki AM uniquely contributes to it, more extensively and clearly in the updated Discussion and briefly in the introduction (L50, 226-229).

The authors claim “deep” and “real-time”. This also seems vastly overstated: 7 mm does not qualify as deep, and the approach appears to be as real-time as other techniques, considering the authors need to use SVD filtering. (Anecdotally, the lengthy description of rather standard SVD filtering could be shortened and make room for more valuable information). In that respect, I am also not convinced of the value of creating a new (and rather uninformative) name for the translation from 2D to 3D of an established technique, although I fully understand the marketing value. If I have overlooked novelty aspects with respect to the pulsing schemes, please make them explicit. The title (and some claims in the paper) do not seem to reflect the work appropriately.

Thank you for the comment. In in vivo molecular and cellular imaging, which has been dominantly performed with optical methods, we think 7-10 mm is deep, and this is important to state for general readers (who come from outside the ultrasound field). We have clarified in the main text that “deep” refers specifically to molecular and cellular imaging (lines 14-17). SVD filtering was applied to BURST processing but not to real-time nonlinear imaging. We believe the name “Takoyaki” effectively provides a good visual representation of how we adapted 3D MLT to a multiplexing system, and the community has responded positively to the name.

With respect to the imaging depth, I believe I understand better the authors. The point, however (and this starts to feel quite redundant), is that either the paper is positioned as a potential clinical or preclinical tool for humans, and this is superficial, or it is positioned as a tool for small animal imaging, and the depth is fine. I have no issue with either, but the authors must make a choice.

As we mentioned in our response to the first and other similar comments, to clarify that this first publication on Takoyaki imaging is focused on small-animal/pre-clinical studies, we have revised the manuscript that might be interpreted as implying clinical applications of the current technique. (Please refer to our response to the first comment for the updated sections)

Reviewer #3 (Remarks to the Author):

The revisions have made clear that the main contribution is the development of a volumetric ultrasound imaging approach ("takoyaki" pulse sequencing) that uses multiline transmission on a multiplexed matrix array to achieve sufficient pressure for nonlinear gas vesicle (and possibly other related contrast agents) imaging. This enables "real-time" (~1 fps depending on settings) visualization of nanoparticle transport in mouse brain ventricles and tumor visualization. The contrast agent (GVs) and nonlinear imaging schemes have been demonstrated previously in planar imaging. The technical advance is showing how to perform multiline transmit imaging on a multiplexed matrix array (compared to the cited fully-sampled matrix array approaches published

by others previously) for nonlinear imaging. While the advance to volumetric imaging is somewhat incremental, I see this paper being impactful as the work that shows the most clinically relevant (3D) imaging technique if nonlinear contrast agents (esp. gas vesicles) become more common.

The preclinical work should be clear in the abstract - add "mouse" to "dynamics of fluid motion in brain ventricles".

As suggested, we have added “mouse” to “dynamics of fluid motion in brain ventricles” in the abstract.

The authors have otherwise addressed my concerns with the manuscript.

Reviewer #3 (Remarks on code availability):

Code was reviewed previously and satisfactory

References

1. Vert, M. *et al*. Transcranial brain-wide functional ultrasound and ultrasound localization microscopy in mice using multi-array probes. *Sci. Rep.* **15**, 12042 (2025).
2. Stringer, C. & Pachitariu, M. Computational processing of neural recordings from calcium imaging data. *Curr. Opin. Neurobiol.* **55**, 22–31 (2019).
3. Errico, C. *et al*. Transcranial functional ultrasound imaging of the brain using microbubble-enhanced ultrasensitive Doppler. *NeuroImage* **124**, 752–761 (2016).
4. Tiran, E. *et al*. Transcranial Functional Ultrasound Imaging in Freely Moving Awake Mice and Anesthetized Young Rats without Contrast Agent. *Ultrasound Med. Biol.* **43**, 1679–1689 (2017).
